# Ohmic Contact Resistance in Wide-Bandgap and Ultrawide-Bandgap Power Semiconductors: From Fundamental Physics to Interface Engineering

**DOI:** 10.3390/ma19071424

**Published:** 2026-04-02

**Authors:** Martin Weis

**Affiliations:** Faculty of Electrical Engineering and Information Technology, Slovak University of Technology, Ilkovicova 3, 841 04 Bratislava, Slovakia; martin.weis@stuba.sk

**Keywords:** ohmic contacts, wide-bandgap semiconductors, specific contact resistivity

## Abstract

Ohmic contact resistance is a persistent and increasingly dominant bottleneck limiting the practical performance of wide-bandgap (WBG) and ultrawide-bandgap (UWBG) power semiconductor devices. This review provides a comprehensive and comparative treatment of specific contact resistivity ($\rho_{c}$) phenomena across five material systems—4H-SiC, GaN, β-Ga_2_O_3_, AlN/AlGaN, and diamond—spanning fundamental contact physics, characterization methodology, material-specific state of the art, device context, and advanced engineering strategies. A semi-empirical scaling analysis establishes that the minimum achievable $\rho_{c}$ increases by approximately one order of magnitude per 0.8–1.0 eV increase in bandgap, arising from the interplay of Fermi-level pinning, increasing carrier effective mass, and decreasing achievable near-surface doping concentration. The best demonstrated $\rho_{c}$ values range from ~3 × 10^−8^ Ω·cm^2^ for GaN epitaxially regrown contacts to ~8 × 10^−5^ Ω·cm^2^ for direct AlN metallization. The transition from alloyed to regrown contacts in GaN—delivering two orders of magnitude improvement—is identified as the paradigm model for UWBG contact development, with β-Ga_2_O_3_ most immediately positioned to follow this trajectory. Key challenges include the absence of *p*-type doping in β-Ga_2_O_3_, near-complete Fermi-level pinning in AlN, and the unsolved shallow-donor problem in diamond. Recommendations for standardized $\rho_{c}$ measurement protocols and priority research directions are presented.

## 1. Introduction

Power semiconductor devices process more than 80% of all generated electricity worldwide, and conduction losses in power conversion systems remain a persistent, technologically addressable fraction of total system inefficiency [1,2]. The electrification of transportation, expansion of renewable energy infrastructure, and proliferation of data centers are driving unprecedented growth in demand for high-efficiency power converters, placing enormous pressure on device performance to reduce conversion losses at every stage of the energy chain. Against this backdrop, the materials science and device physics communities face an urgent mandate to push semiconductor power device performance beyond the limits imposed by silicon, which—after decades of steady incremental improvement—is now broadly considered to be approaching its theoretical performance ceiling [3].

**Figure 1:**
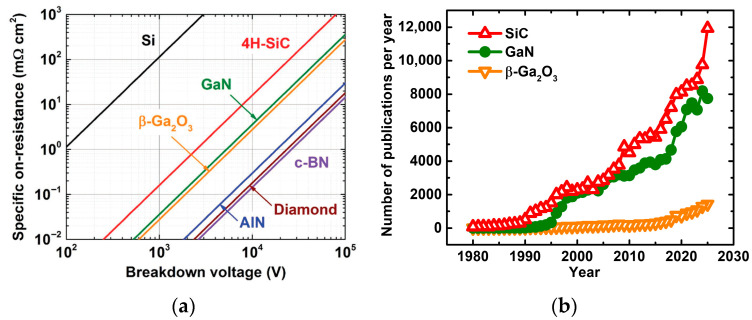
(**a**) Contours of constant Baliga figure-of-merit (BFOM ∝ $\mu E_{c}^{3}$) for conventional, WBG, and UWBG semiconductors, drawn on a log–log specific on-resistance versus breakdown voltage plot. Contour lines represent (from lowest to highest BFOM): Si (reference), 4H-SiC (~340× Si), GaN (~870× Si), β-Ga_2_O_3_ (~3200× Si), cubic boron nitride (ε-BN), AlN (~40,000× Si), and diamond (~25,000× Si). This is the figure-of-merit of interest for low-frequency unipolar vertical power switches; the lower right region represents higher BFOM, hence higher performance (adapted from Tsao et al. [6]). (**b**) Number of publications published on selected WBG and UWBG semiconductors (Web of Science Core Collection database).

Wide-bandgap (WBG) semiconductors—most prominently silicon carbide (SiC) and gallium nitride (GaN)—have emerged as the leading successors to silicon in demanding power conversion applications, offering substantially higher critical electric field strengths, elevated operating temperatures, and faster switching capabilities. The Baliga figure of merit (BFOM $=\varepsilon_{s}\mu E_{c}^{3}$, i.e., product of dielectric constant, $\varepsilon_{s}$; mobility, μ; and critical electric field, $E_{c}$), which quantifies the trade-off between drift layer resistance and blocking capability, exceeds silicon by approximately 340× for 4H-SiC and 870× for GaN [1]. Commercial SiC Schottky diodes and MOSFETs already displace silicon in 650–3300 V automotive, industrial, and photovoltaic applications, and GaN-based high-electron-mobility transistors (HEMTs) have established a strong presence in sub-600 V lateral switching and radio-frequency power amplification [4,5]. An emerging class of ultrawide-bandgap (UWBG) semiconductors—including gallium oxide (β-Ga_2_O_3_), aluminum nitride (AlN), high-aluminum-content AlGaN alloys, and diamond—promises a further order-of-magnitude improvement in critical field, with theoretical BFOM values reaching 3000–40,000× silicon, if the associated materials and processing challenges can be overcome [6,7], as illustrated in Figure 1a. These unique properties drive the growing research interest, as shown in Figure 1b.

Despite compelling material properties, the full theoretical potential of WBG and UWBG devices remains elusive in practice. One of the most persistent and underappreciated obstacles is the resistance associated with metal–semiconductor ohmic contacts. In any unipolar power device—the category that encompasses most of the high-frequency and high-efficiency switches—the total on-state resistance, *R_on_*, is the sum of contributions from the channel, drift region, substrate, and contact regions. As drift-layer and channel engineering have matured, the relative contribution of contact resistance to *R_on_* has grown steadily; in lateral GaN HEMTs and emerging β-Ga_2_O_3_ transistors, contact and access resistance together account for a dominant fraction of the total on-resistance budget [8,9]. Reducing specific contact resistivity, $\rho_{c}$—the material- and geometry-normalized metric of contact quality—is, therefore, not merely an incremental processing concern but a fundamental bottleneck governing how closely fabricated devices approach the theoretical performance ceiling of their constituent materials.

The physical origin of this challenge is rooted in the electronic structure of WBG and UWBG semiconductors. Forming a truly ohmic contact requires either a vanishingly small Schottky barrier height or sufficiently high carrier concentration at the interface to enable efficient quantum-mechanical tunneling through the residual barrier. Both conditions are difficult to realize simultaneously in wide-bandgap materials. The large bandgap inherently limits the range of metal work functions that can achieve a barrier-free interface in the Schottky–Mott picture, while Fermi-level pinning—driven by interface states whose density and energy distribution vary characteristically across material systems—further constrains the achievable barrier height independently of metal choice [10]. On the doping side, WBG and UWBG materials suffer from high dopant activation energies (aluminum acceptors in SiC at ~200 meV; Mg acceptors at ~170 meV), the absence of viable *p*-type doping in β-Ga_2_O_3_, or near-complete Fermi-level pinning that renders metal selection effectively irrelevant in AlN. The result is that achieving specific contact resistivities in the range of 10^−6^ Ω·cm^2^ or below—a target broadly required for next-generation high-current devices—remains an open and actively contested research problem across virtually all WBG and UWBG material families [11].

The body of literature addressing contact resistance in WBG and UWBG semiconductors has expanded considerably over the past decade, yet it remains fragmented across material-specific communities that do not always communicate fluidly. SiC contact engineering has a long and sophisticated history rooted in the power semiconductor community; GaN contact research is heavily intertwined with III-nitride epitaxy and HEMT physics; and UWBG contact work is still largely exploratory. Existing reviews have either focused on individual material systems or addressed the broader landscape of WBG device physics without dedicating systematic attention to the contact resistance problem in its own right [12,13,14,15]. This review aims to fill that gap by providing a comprehensive and comparative treatment of contact resistance phenomena across five material systems, connecting fundamental physics, characterization methodology, material-specific state of the art, device-level implications, and emerging engineering strategies. The coverage spans approximately 2015–2025, drawing on foundational earlier work where necessary.

This review is organized as follows. Section 2 develops the fundamental physics of metal–semiconductor contacts in WBG materials and reviews the principal characterization techniques and their limitations. Section 3, Section 4, Section 5 and Section 6 address the material-specific state of the art for 4H-SiC, GaN, β-Ga_2_O_3_, and UWBG frontier materials (AlN/AlGaN and diamond). Section 7 provides a cross-material comparative analysis and benchmark. Section 8 situates contact resistance within the device performance and reliability context and surveys advanced engineering strategies. Section 9 concludes with a synthesis and forward-looking perspective.

## 2. Contact Physics and Characterization Methods

### 2.1. Schottky Barrier Formation and Fermi-Level Pinning

When a metal is brought into close contact with a semiconductor, the energy band alignment that develops at the interface determines whether the junction is rectifying (Schottky) or ohmic in character. The idealized Schottky–Mott model predicts the electron barrier height as $\varphi_{Bn}=\varphi_{m}-\chi_{s}$, where $\varphi_{m}$ is the metal work function, and $\chi_{s}$ is the semiconductor electron affinity, with the complementary hole barrier given by $\varphi_{Bh}=E_{g}-\varphi_{Bn}$. This picture implies continuous tunability of barrier height through metal selection—a strategy that provides useful leverage in silicon and germanium but fails progressively as bandgap increases.

The failure of the Schottky–Mott model in WBG and UWBG materials arises from Fermi-level pinning: a high density of interface states within the semiconductor bandgap—originating from surface reconstruction, dangling bonds, metal-induced gap states (MIGS), or defect complexes formed during metallization—forces the Fermi level to an approximately fixed energy at the interface regardless of the metal used [8,16]. The severity of pinning is quantified by the pinning parameter $S={\partial \varphi }_{Bn}/{\partial \varphi }_{m}$; S=1 is the ideal Schottky–Mott limit, while S→0 represents complete pinning. For practical WBG semiconductors, S ranges from ~0.5 to 0.7 in β-Ga_2_O_3_ (moderate pinning) and ~0.1 to 0.2 in AlN and diamond (near-complete pinning) [10,17]. The consequence for contact engineering is direct: even if an ideal barrier-reducing metal could be identified, the actual achievable barrier height is controlled primarily by interface chemistry rather than metal work function, placing interface preparation and defect engineering at the center of contact optimization.

In UWBG materials, Fermi-level pinning has an additional fundamental consequence. For AlGaN with an Al fraction above ~0.5, the electron affinity falls below ~2.1 eV—lower than the work function of any stable metal under ambient conditions (~3.5 eV for cesium, the lowest practical value)—making barrier-free *n*-type ohmic contact in the Schottky–Mott picture physically impossible. The minimum achievable barrier height in such materials is determined entirely by the pinning position, and even the most favorable S values reported for III-nitrides (~0.2–0.3) cannot bring $\varphi_{Bn}$ below ~1.0–1.5 eV for high-Al compositions [18]. This analysis establishes a fundamental physical constraint that pure metallization optimization cannot overcome and that motivates the structural and epitaxial contact engineering strategies discussed in later sections.

The physical origin of pinning strength varies significantly across WBG/UWBG materials. In AlN, severe pinning (S ≈ 0.15–0.3) arises from: (i) high metal-induced gap state (MIGS) density due to poor dielectric screening ($\varepsilon_{r}$ ≈ 8.5), (ii) unfavorable charge neutrality level (CNL) position ~1.6 eV above the conduction band minimum, and (iii) a reactive surface chemistry with native Al_2_O_3_ formation introducing additional interface states [16,17]. In contrast, β-Ga_2_O_3_ exhibits moderate pinning (S ≈ 0.6) due to: (i) lower MIGS density from better screening ($\varepsilon_{r}$ ≈ 10), (ii) a CNL positioned closer to the conduction band, and (iii) a self-passivating oxide surface with inherently lower defect density [19]. The relationship between dielectric constant and MIGS density—predicted by Mönch’s model as $D_{MIGS}$ ∝ 1/$\varepsilon_{s}$—explains why pinning generally strengthens with an increasing bandgap in the nitride family (GaN S ≈ 0.3 → AlN S ≈ 0.15), while the oxide β-Ga_2_O_3_ maintains relatively weak pinning despite its large bandgap.

### 2.2. Valence Band Structure Impact on p-Type Tunneling

The *p*-type contact resistance penalty extends beyond deeper acceptor levels to include a fundamental quantum mechanical disadvantage: the valence band effective mass structure reduces tunneling probability in the field emission regime. The barrier transmission coefficient, T ∝ exp(−2κd) where $\kappa \;=\;(1/\hbar )\sqrt{2m_{\mathrm{tunnel}}^{*}\;\varphi_{B}}$, depends critically on the tunneling effective mass.

Unlike the conduction band, which is typically a single parabolic band with $m_{e}$ ~ 0.2–0.4 $m_{0}$, the valence band comprises multiple sub-bands (heavy hole, light hole, and split-off) with distinct effective masses. In wurtzite GaN/AlN, the heavy hole band exhibits $m_{hh,⊥}$ ~ 1.4–2.0 $m_{0}$ perpendicular to the c-axis—4–10× heavier than conduction band electrons. The heavy hole band sits at the valence band maximum and possesses the highest density of states, thus carrying the majority of hole current despite having the poorest tunneling transmission.

Quantitatively, for identical barrier heights, $\varphi_{B}$; doping levels; and depletion widths, the tunneling transmission ratio scales as $T_{p}/T_{n}\;\sim \;\exp[-K\;\times \;(\sqrt{m_{hh}}-\;\sqrt{m_{e}})]$, where K depends on $\varphi_{B}$ and the electric field. For typical WBG parameters with $m_{hh}/m_{e}$ ~ 4–8, this yields $T_{p}$ ~ 0.01–0.1 × $T_{n}$, i.e., 1–2 orders of magnitude transmission penalty independent of doping considerations. Combined with typically 5–10× lower achievable *p*-type doping ($N_{A,max}<\;N_{D,max}$ to deeper acceptors and lower solubility), the cumulative effect explains the consistent 1.5–2.5 order-of-magnitude $\rho_{c,p}/\rho_{c,e}$ ratio observed across WBG semiconductors, as illustrated in Table 1.

Additional contributions include: (i) anisotropic tunneling in wurtzite structures where vertical devices utilize the heaviest mass direction ($m_{hh,⊥}$), (ii) shorter hole de Broglie wavelength increasing interface roughness scattering, and (iii) often larger *p*-type barriers ($\varphi_{Bp}=\;E_{g}-\;\chi \;-\;\varphi_{m}$) for the same metal work function.

### 2.3. Transport Mechanisms and the Specific Contact Resistivity

**Figure 2:**
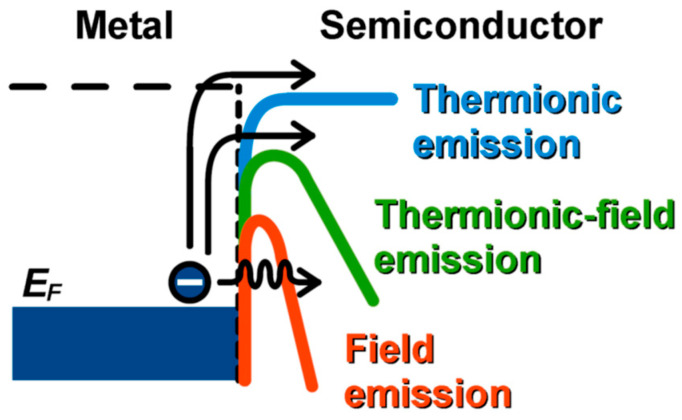
Schematic view of three major charge carrier transport regimes at metal–semiconductor interface.

Three carrier transport regimes govern metal–semiconductor contact behavior, each dominant in different combinations of barrier height, semiconductor doping concentration, and temperature [20,21], as illustrated in Figure 2.

Thermionic emission (TE) dominates at low doping and elevated temperatures: carriers surmount the barrier classically, and current density follows $J=AT^{2}\exp\left(-q\varphi_{Bn}/kT\right)\left[\exp\left(qV/kT\right)-1\right]$, where A is the effective Richardson constant. TE contacts are inherently rectifying for any finite barrier; achieving ohmic behavior in this regime requires $\varphi_{Bn}$ to be negligible relative to thermal energy, kT, which is generally unachievable in WBG systems.

Field emission (FE), or direct quantum-mechanical tunneling through the barrier, becomes dominant when heavy doping narrows the depletion width to the point where the tunneling probability is appreciable. In this regime, the specific contact resistivity scales as $\rho_{c}\propto \exp\left(C\varphi_{Bn}/\sqrt{N_{D}}\right)$, where $C=(4\pi /h)\sqrt{2m\varepsilon_{s}}$ incorporates the material-dependent effective mass m and permittivity $\varepsilon_{s}$. While C varies by approximately a factor of 2 across the materials reviewed (from 0.66 for diamond to 1.26 for SiC, normalized to GaN = 1), the exponential dependence on barrier height, $\varphi_{Bn}$, dominates the scaling behavior. With $\varphi_{Bn}\approx \;0.3-0.4\;\times \;E_{g}$, due to Fermi-level pinning and achievable doping, $N_{D}$, decreasing with increasing bandgap, the semi-empirical trend of $\rho_{c}$ increasing by approximately one order of magnitude per 0.8–1.0 eV bandgap increase emerges from the combined effects. This expression is the central result for WBG contact engineering: it quantifies the simultaneous requirements for low barrier height and high near-surface doping. Since both $\varphi_{Bn}$ (through pinning constraints) and effective mass increase with bandgap, while achievable $N_{D}$ is limited by dopant solubility and activation energy, a fundamental penalty in minimum achievable $\rho_{c}$ with increasing $E_{g}$ emerges from straightforward physics—approximately one order of magnitude per 0.8–1.0 eV increase in bandgap.

Thermionic-field emission (TFE) is the intermediate regime ($E_{00}\approx kT$, where $E_{00}=\left(q\hbar /2\right){\left(N_{D}/m^{*}\varepsilon_{s}\right)}^{1/2}$), with carriers tunneling near the top of the barrier where it is thinnest. This mechanism frequently governs SiC *p*-type contacts and as-deposited or partially annealed contacts in other WBG systems. Temperature-dependent characterization that identifies the operative regime is, therefore, essential for physically meaningful barrier height extraction and contact optimization.

The transition between transport regimes is governed by the characteristic tunneling energy, $E_{00}\;=\;(q\hbar /2)\sqrt{N_{D}/(m^{*}\varepsilon_{0}\varepsilon_{r})}$, relative to thermal energy, kT. The TFE→FE transition occurs when $E_{00}\approx \;kT$, corresponding to a critical doping concentration of $N_{D,transition}$ ≈ 1.2 × 10^18^ × ($m^{*}$/$m_{0}$) × $\varepsilon_{r}$.

Table 2 summarizes transition concentrations for the reviewed materials. Several observations emerge:

(1) GaN regrowth contacts ($N_{D}$ > 5 × 10^20^ cm^−3^) operate firmly in the FE regime with $E_{00}$ ~ 120 meV ≫ kT, explaining the exceptional $\rho_{c}$ ~ 3 × 10^−8^ Ω·cm^2^.
(2) Conventional SiC contacts ($N_{D}$ ~ 2 × 10^19^ cm^−3^) remain in the TFE regime where $\rho_{c}$ retains exponential temperature dependence.
(3) AlN’s DX-center-limited doping ($N_{D}$ ~ 10^18^ cm^−3^) barely reaches the transition criterion, leaving contacts in the unfavorable TE regime.
(4) β-Ga_2_O_3_ epitaxial contact layers ($N_{D}$ ~ 10^20^ cm^−3^) approach the FE regime despite a larger bandgap than SiC, positioning the material favorably for future optimization.

**Table 2:** Transport regime classification for WBG/UWBG ohmic contacts at 300 K.

Material	$m^{*}/m_{0}$	$\varepsilon_{r}$	$N_{D,transition}$	$\mathrm{Typical}\;N_{D}$	$E_{00}$ (meV)	Regime	$\rho_{c}$ Impact
Si (ref.)	1.08	11.7	1.5 × 10^19^	>10^20^	150	FE	$\rho_{c}<$ 10^−8^
SiC (implant)	0.40	10.0	4.8 × 10^18^	2 × 10^20^	55	TFE	$\rho_{c}$ ~ 10^−6^
GaN (regrown)	0.20	9.0	2.2 × 10^18^	>5 × 10^20^	120	FE	$\rho_{c}$ ~ 10^−8^
GaN (conventional)	0.20	9.0	2.2 × 10^18^	2 × 10^19^	36	TFE	$\rho_{c}$ ~ 10^−6^
Ga_2_O_3_ (epi)	0.28	10.0	3.4 × 10^18^	10^20^	90	TFE/FE	$\rho_{c}$ ~ 10^−6^
AlN	0.30	8.5	3.1 × 10^18^	~10^18^	26	TE/TFE	$\rho_{c}$ ~ 10^−4^
Diamond (p)	0.70	5.5	4.6 × 10^18^	>10^21^	200	FE	$\rho_{c}$ ~ 10^−6^

The specific contact resistivity $\rho_{c}=\;{\left(\partial J/\partial V\right)}^{-1}{|}_{V=0}$ (Ω·cm^2^) is independent of contact geometry, making it the standard cross-material comparison metric. It relates to the contact resistance, $R_{c}$ (Ω), through the contact area and to the transfer length, $L_{T}=\sqrt{{\rho_{c}/R}_{sh}}$—the characteristic length over which current flows into or out of the contact—through $\rho_{c}\;=\;R_{sh}L_{T}^{2}$, where $R_{sh}$ is the semiconductor sheet resistance beneath the contact [22]. Current does not flow uniformly across the contact footprint but concentrates within the $L_{T}$ of the contact edge; failure to account for this non-uniformity in contact test structure design leads to systematic errors in $\rho_{c}$ extraction.

The achievable range of $\rho_{c}$ spans many orders of magnitude across the materials reviewed: from below 10^−8^ Ω·cm^2^ for silicon CMOS and best-case GaN regrown contacts to ~10^−5^–10^−4^ Ω·cm^2^ for direct contacts to AlN and diamond H-surface. The systematic one-to-two-order-of-magnitude offset between *n*-type and *p*-type contact performance—observed across SiC, GaN, and diamond—reflects the combination of a larger hole effective mass, deeper acceptor ionization energies, and typically lower achievable *p*-type surface concentrations.

### 2.4. Contact Resistance Characterization Methods

**Figure 3:**
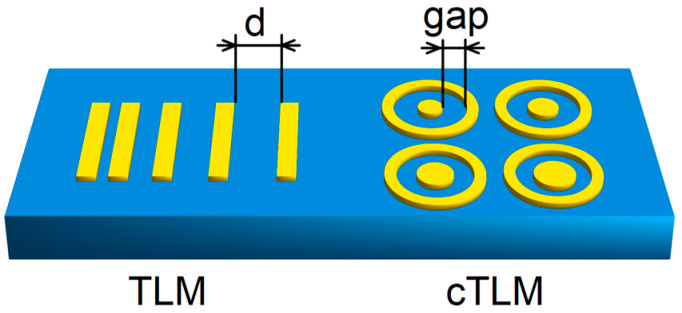
Schematic view of the electrode geometry used for Transfer Length Method (TLM) and circular TLM (cTLM). The parameter d represents the varying inter-electrode spacing in the linear TLM, while *gap* denotes the radial separation between the inner disk and outer ring electrodes in the cTLM structures.

The Transfer Length Method (TLM) is by far the most widely used technique for $\rho_{c}$ measurement in semiconductor device research and remains the workhorse of WBG contact development [23]. In the linear TLM, a series of contacts with progressively increasing gap spacings, d, are fabricated on a semiconductor layer; total resistance, $R_{total}=2R_{c}+R_{sh}d/W$, plotted against d, yields $\rho_{c}$, $R_{sh}$, $R_{c}$, and $L_{T}$ simultaneously from the slope and y-intercept. Despite conceptual simplicity, TLM carries systematic errors amplified in WBG systems. Mesa isolation—required to prevent substrate leakage—introduces the risk of sidewall conduction and etch-induced damage artifacts beneath the contacts [24]. The sheet resistance beneath the contacts may differ substantially from inter-contact, $R_{sh}$, when annealing forms reactive metallurgical phases (silicides and nitrides) that modify the near-surface semiconductor layer [25]. High $R_{sh}$ in UWBG materials (>10^4^ Ω/sq. for moderately doped β-Ga_2_O_3_) makes the y-intercept determination geometrically inaccurate when all gap spacings exceed $L_{T}$. The non-linearity of partially ohmic contacts at a small measurement bias distorts the extracted $\rho_{c}$ toward the value at measurement voltage rather than at zero bias [20]. The circular TLM (cTLM) circumvents the need for mesa isolation by confining current radially between concentric contacts, making it especially valuable for UWBG materials where etch processes are immature and damage-induced leakage is a concern [26], as shown in Figure 3.

Four-probe Kelvin contact measurements—distinct from the four-probe van der Pauw method used for semiconductor sheet resistance characterization—provide a direct assessment of metal/semiconductor interface resistance independent of spreading resistance contributions. In Kelvin contact structures, voltage-sensing probes are placed inside the contact metallization, measuring the voltage drop directly across the interface while current is forced through separate outer probes. This geometry eliminates series resistance from current spreading in the semiconductor, isolating the true contact resistivity, $\rho_{c}$. Four-probe Kelvin measurements serve as an indispensable cross-check on TLM results—particularly for as-deposited contacts or in systems where sub-contact sheet resistance modification is suspected [27]. The cross-bridge Kelvin resistor (CBKR) structure extends this approach for small-area contacts, where $L_{T}$ is comparable to contact dimensions, and the TLM one-dimensional current flow assumption fails [27]. Temperature-dependent I–V (I–V–T) characterization is indispensable for identifying the operative transport regime and extracting activation energies that distinguish TE ($E_{a}=\;q\varphi_{Bn}$, strong T-dependence), TFE ($E_{a}$ ~ 0.1–0.4 eV, intermediate), and FE ($E_{a}\;<$ 0.1 eV, weak T-dependence) mechanisms. Systematic I-V-T measurements over 300–500 K enable physically self-consistent barrier height extraction and predict high-temperature contact performance—critical for power electronics operating above 200 °C (Section 8.2). [28]. Scanning probe techniques—conductive AFM, Kelvin probe force microscopy (KPFM), and scanning spreading resistance microscopy (SSRM)—provide local mapping of contact properties at nanometer resolution, revealing spatial barrier height inhomogeneity that underlines macroscopic current transport [29].

A significant and underappreciated source of inter-laboratory scatter in reported $\rho_{c}$ values is inconsistency in surface preparation before metallization. For β-Ga_2_O_3_, re-oxidation in ambient air within minutes of HF treatment shifts $\rho_{c}$ by factors of 2–10; for SiC, native oxide regrowth between cleaning and deposition chamber entry meaningfully impacts Ni silicide formation kinetics. The silicon CMOS community addressed this through formally standardized substrate-specific cleaning protocols (RCA, Shiraki) and SEMI-standardized test structure geometries. No equivalent standard exists for any WBG material system, and the resulting one-to-two-order-of-magnitude scatter in reported $\rho_{c}$ within each material system—far beyond genuine process variation—substantially reflects measurement methodology inconsistency [22,23]. Adoption of harmonized protocols specifying cleaning chemistry and timing, TLM geometry rationale, bias linearity verification, and temperature-dependent supplementary measurements would dramatically improve the field’s ability to assess genuine technological progress.

## 3. Silicon Carbide (SiC)

### 3.1. Material Context and Commercial Status

Silicon carbide is simultaneously the most physically well-characterized, the most commercially mature, and the material whose contact technology has undergone the longest and most systematic development among WBG semiconductors. Commercial 4H-SiC Schottky barrier diodes have been available since the early 2000s, and SiC power MOSFETs entered volume production during the following decade, driven by demand from automotive inverters, photovoltaic converters, and industrial motor drives [30,31]. As of the mid-2020s, 4H-SiC devices dominate the 650–3300 V power switching market segment, where switching frequency, thermal performance, and efficiency justify the cost premium over silicon IGBTs.

Among the more than 200 known SiC polytypes, only 4H-SiC (and to a lesser extent, 6H-SiC) is relevant to power device fabrication. 4H-SiC is strongly preferred due to its wider bandgap (3.26 eV vs. 3.03 eV for 6H-SiC), higher and isotropic electron mobility (~1000 cm^2^/V·s in lightly doped epilayers perpendicular to c-axis), and superior availability of high-quality homoepitaxial substrates grown by sublimation [15]. The critical electric field of ~2.5 MV/cm yields a theoretical minimum specific on-resistance of ~0.4 mΩ·cm^2^ at 1200 V. In state-of-the-art 1200 V SiC MOSFETs with measured $R_{on}$ of 1–5 mΩ·cm^2^, source and drain ohmic contacts together contribute 10–30% of the total, making contact resistance a primary device performance parameter—not a secondary processing detail [32]. The substantially lower hole mobility (~115 cm^2^/V·s) compared to electron mobility has direct and important consequences for the comparative difficulty of *n*-type versus *p*-type contact formation, and the two problems are discussed separately.

### 3.2. N-Type Ohmic Contacts: Nickel Silicide System

Nickel has emerged as the dominant and most thoroughly understood *n*-type contact metallization for 4H-SiC after more than three decades of systematic investigation [33]. As-deposited Ni on *n*-type SiC exhibits Schottky behavior with barrier height ~1.5 eV, entirely unsuitable for ohmic applications. The transformation to ohmic behavior is achieved by rapid thermal annealing (RTA) at 900–1050 °C, during which Ni reacts with SiC to form nickel silicide (primarily Ni_2_Si) and releases carbon as a graphitic surface layer via: 2Ni + SiC → Ni_2_Si + C [34]. The Ni_2_Si phase has a work function of ~4.7 eV and forms an interface that supports efficient tunneling transport, yielding $\rho_{c}$ in the range of 1–5 × 10^−6^ Ω·cm^2^ for *n*-type concentrations of 10^18^–10^19^ cm^−3^ [35].

The mechanistic origin of barrier height reduction during Ni_2_Si formation remains an active area of investigation, with three competing hypotheses:

(1) Carbon segregation model: The silicidation reaction 2Ni + SiC → Ni_2_Si + C releases excess carbon that may segregate to the Ni_2_Si/SiC interface or grain boundaries. TEM and Raman studies have detected carbon-rich regions and graphitic signatures at some interfaces [34,36], suggesting semi-metallic carbon clusters could provide conductive pathways or enhance local tunneling probability. However, this mechanism is inconsistent: not all high-performance contacts show carbon accumulation, and some exhibit carbon depletion at interfaces.
(2) Silicon vacancy engineering: Silicidation creates silicon vacancies ($V_{Si}$) in the near-interface SiC region. As $V_{Si}$ acts as a shallow donor in 4H-SiC ($E_{d}$ ~ 0.6 eV below CBM), high vacancy concentrations could form a heavily doped interfacial layer, reducing depletion width [36,37]. DLTS and positron annihilation measurements confirm increased $V_{Si}$ concentration near contacts. The challenge is that required concentrations (>10^19^ cm^−3^) appear unrealistically high, and $V_{Si}$’s relatively deep ionization energy questions room-temperature effectiveness.
(3) Work function matching and interface state reduction: Ni_2_Si has the work function $\varphi_{m}$ ~ 4.7 eV, yielding a lower theoretical Schottky barrier ($\varphi_{Bn,ideal}$ $=\;\varphi_{m}-\;\chi$ ~ 1.4 eV) than pure Ni ($\varphi_{m}$ ~ 5.1 eV). Additionally, the specific Ni_2_Si/SiC atomic interface may exhibit reduced MIGS density compared to as-deposited Ni/SiC. I-V-T measurements typically extract $\varphi_{Bn}$ ~ 0.6–0.9 eV [35,38], consistent with work function considerations and moderate Fermi-level pinning (S ~ 0.3).

These mechanisms are not mutually exclusive—all three may contribute with relative importance depending on annealing conditions (temperature, atmosphere, and time). The lack of consensus reflects characterization challenges in isolating nm scale interface effects and process sensitivity, where subtle variations in annealing protocol produce different interface structures. Atomic-resolution STEM-EELS mapping and operando barrier measurements are needed to definitively resolve the relative contributions.

Proposed contributions include Ni_2_Si work function alignment with the SiC conduction band, the creation of a locally nitrogen-enriched interfacial SiC layer through carbon extraction (which increases local carrier concentration), and the formation of a spatially inhomogeneous interface with locally thin tunneling barriers at silicide grain boundaries [36]. This mechanistic uncertainty is reflected in the sensitivity of the process to annealing conditions: temperatures below ~850 °C leave the contact partially reacted with high $\rho_{c}$ and non-linear I–V characteristics; temperatures above ~1100 °C promote higher silicide phases (NiSi and NiSi_2_) and morphological agglomeration, degrading both $\rho_{c}$ and contact uniformity. The optimal annealing window is narrow, typically 950–1000 °C for 30–100 nm Ni films, and must be determined empirically for each metallization thickness and doping level [30].

The mechanistic uncertainty is exacerbated by strong process dependence. Contacts annealed at 950 °C show primarily Ni_2_Si with minimal carbon segregation, while 1050 °C annealing promotes carbon precipitation and may form NiSi at prolonged times. Annealing atmosphere also matters: vacuum promotes Si out-diffusion, creating Si-rich silicides, while N_2_ can introduce nitrogen contamination. This process window complexity means published results span a range of interface structures, making mechanistic comparisons difficult. What is clear is that the Ni_2_Si phase itself is necessary but not sufficient—interface quality, carbon distribution, and defect structure all influence final $\rho_{c}$ within the 10^−7^ to 10^−5^ Ω·cm^2^ range typically reported.

Alternative *n*-type metallization systems have been extensively explored in pursuit of $\rho_{c}$ below 10^−6^ Ω·cm^2^ or improved thermal stability. Titanium forms TiC and TiSi_2_ at lower temperatures (~800–900 °C) but typically shows higher $\rho_{c}$ than optimized Ni contacts at equivalent doping, and its greater oxidation susceptibility limits practical application [39]. Cobalt silicide (CoSi_2_) contacts achieve $\rho_{c}$ values comparable to Ni_2_Si at nitrogen concentrations above 5 × 10^18^ cm^−3^, with improved high-temperature stability reported in some studies. Implantation-assisted contacts—where nitrogen or phosphorus ions are pre-implanted into the contact region before metallization to create a locally very heavily doped zone—have demonstrated $\rho_{c}$ approaching 10^−7^ Ω·cm^2^, an order of magnitude below the Ni silicide baseline, but they require implant activation annealing above 1600 °C that must precede any other device fabrication steps [32,40].

Comparison with alternative silicides provides mechanistic clues. Cobalt silicide (CoSi_2_) contacts achieve $\rho_{c}$ ~ 2 × 10^−6^ Ω·cm^2^ [39]—competitive with Ni_2_Si—despite different work function ($\varphi_{m}$ ~ 4.3 eV), suggesting work function alone does not dominate. Titanium silicide (TiSi_2_, $\varphi_{m}$ ~ 4.1 eV) gives inferior performance despite a lower theoretical barrier, indicating interface state density or morphology plays a critical role. These observations favor a combined mechanism where the silicide phase determines baseline barrier (work function effect), but carbon/vacancy distribution at the specific interface structure modulates tunneling probability.

### 3.3. p-Type Ohmic Contacts

*p*-type ohmic contacts to 4H-SiC represent the primary unresolved contact challenge for bipolar device architectures, including PiN diodes, Bipolar Junction Transistor (BJTs), and CMOS complementary structures. The large aluminum acceptor ionization energy (~200 meV) means only ~1% of incorporated Al atoms contribute free holes at 300 K in lightly doped material, while achieving the surface concentrations above 10^19^–10^20^ cm^−3^ needed for effective tunneling requires implant doses and annealing temperatures at the boundary of practical process capability. Standard *p*-type metallization uses Al-rich Ti/Al alloy or multilayer stacks annealed at 1000–1100 °C, forming mixed carbide and aluminide phases at the interface [41,42]. The best demonstrated $\rho_{c}$ for *p*-type SiC contacts is approximately 10^−5^–10^−6^ Ω·cm^2^ at Al concentrations above 10^20^ cm^−3^—still one to two orders of magnitude above the *n*-type baseline at comparable doping [43]. This asymmetry arises from the combination of a larger hole effective mass (which exponentially reduces tunneling probability in the FE model), deeper acceptor levels requiring higher absolute doping for equivalent carrier concentration, and greater processing difficulty in achieving uniform, defect-free high-Al surfaces.

Contact reliability under sustained high-temperature operation is a critical qualification for automotive and aerospace applications. Ni_2_Si *n*-type contacts show acceptable morphological stability to ~500 °C, with grain coarsening and carbon redistribution observable above this temperature but without immediate degradation of $\rho_{c}$. *p*-type Al-containing contacts are more vulnerable, with Al outdiffusion and oxidation becoming significant above ~400 °C; Ti/Al/Ni/Au multilayer structures with refractory diffusion barriers have improved stability but at the cost of increased process complexity [38,44]. For device applications requiring sustained operation above 300 °C—a key competitive advantage of SiC over silicon—contact stack design must balance $\rho_{c}$ minimization with thermomechanical stability, and neither objective can be optimized independently.

The transition to FE transport in SiC requires $N_{D}$ > 4.8 × 10^18^ cm^−3^. While post-implantation activation can achieve $N_{D}$ ~ 2 × 10^19^ cm^−3^ ($E_{00}$ ~ 55 meV ≈ 2 kT), contacts remain in the TFE regime where $\rho_{c}$ retains temperature dependence and exponential sensitivity to doping variations. This explains the difficulty in surpassing $\rho_{c}$ ~ 10^−6^ Ω·cm^2^ with conventional approaches.

## 4. Gallium Nitride (GaN)

### 4.1. Device Architecture and Contact Engineering Context

GaN’s bandgap (3.4 eV), critical field (~3.3 MV/cm), and electron saturation velocity (>2.5 × 10^7^ cm/s) make it competitive with SiC across several power device figures of merit [13]. The dominant GaN power device architecture—the lateral AlGaN/GaN HEMT—differs fundamentally from the vertical SiC MOSFET in ways that profoundly shape contact engineering requirements. Source and drain ohmic contacts must make an electrical connection to a polarization-induced two-dimensional electron gas (2DEG) confined ~20–30 nm below the semiconductor surface, separated from the metal by an AlGaN barrier layer [45]. This architecture introduces two geometrically distinct contact resistance components: the intrinsic metal–semiconductor interface resistance at the contact footprint and the access resistance arising from current spreading from the 2DEG to the contact region [46]. Both contribute to the source/drain resistance that limits on-state performance, and both must be addressed by contact engineering.

The 2DEG sheet carrier density—typically 0.8–1.5 × 10^13^ cm^−2^—and its associated sheet resistance of ~300–600 Ω/sq. in ungated regions set a minimum access resistance between contact edge and gate that scales with source-gate spacing independently of contact metallurgy. The transfer length $L_{T}$ at a GaN HEMT contact is typically 0.5–2 μm, comparable to or larger than the contact length in aggressively scaled devices; proper consideration of $L_{T}$ in contact geometry design is essential and is frequently neglected in the literature, leading to contacts that are geometrically larger than necessary and that obscure the intrinsic $L_{T}$ value [47]. The substrate choice—GaN-on-Si for cost-sensitive 600–650 V applications, GaN-on-SiC for high-frequency and high-power-density use—affects thermal management but not fundamentally the contact engineering approach for lateral HEMTs. Vertical GaN devices (Current Aperture Vertical Electron Transistor (CAVETs), FinFETs, and trench MOSFETs) enabled by freestanding GaN substrates require ohmic contacts to *n*-type drift layers and, in some architectures, to *p*-type current-blocking layers, introducing contact challenges analogous to SiC [48].

The AlGaN/GaN heterostructure relies fundamentally on polarization effects: spontaneous polarization ($P_{SP}$) intrinsic to the wurtzite structure and piezoelectric polarization ($P_{PE}$) from strain create a polarization discontinuity at the AlGaN/GaN interface. For Al_x_Ga_(1−x)_N on GaN, the total polarization reaches $P_{total}$ ~ −0.11 C/m^2^ for x = 0.7, corresponding to a bound sheet charge of $\sigma_{pol}$ ~ 7 × 10^13^ cm^−2^—comparable to typical 2DEG densities.

Critically for contact physics, this polarization also affects contacts to the AlGaN barrier layer. The negative surface charge (Ga-polar growth) creates upward band bending at the free surface with electric field $E_{pol}$ ~ −1.5 MV/cm. This pre-existing field modifies the contact barrier height independent of metal work function, $\varphi_{Bn,\mathrm{eff}}\;=\;\varphi_{Bn,\mathrm{conventional}}\;+\;\Delta V_{pol}$, where $\Delta V_{pol}$ is ~ 0.2–0.4 eV for typical high-Al-content barriers. Additionally, the polarization field creates a more triangular (vs. parabolic) barrier profile, modifying the effective tunneling distance. This explains why contacts to high-Al-content AlGaN systematically exhibit higher barriers ($\varphi_{Bn}$ ~ 1.4–1.6 eV) than contacts to GaN ($\varphi_{Bn}$ ~ 0.8–1.0 eV) using identical metals.

### 4.2. Conventional Alloyed Contacts: Ti/Al-Based Metallization

Conventional ohmic contact formation in AlGaN/GaN HEMTs uses metal stacks based on titanium and aluminum—most commonly Ti/Al/Ni/Au or variants with Mo or Nb as diffusion barrier—annealed at 800–900 °C [49]. Upon annealing, Ti reacts with GaN to form TiN while releasing nitrogen, locally creating nitrogen vacancies that act as shallow donors and dramatically increase the near-surface electron concentration. Al simultaneously interdiffuses toward the interface, and the net result is a heavily doped, disordered interfacial zone with quasi-metallic conductivity that provides efficient tunneling contact to the 2DEG [50]. This approach reliably achieves $\rho_{c}$ in the range of 5 × 10^−7^ to 5 × 10^−6^ Ω·cm^2^ under optimized conditions and represents the industry baseline for most current GaN HEMT manufacturing.

However, alloyed contacts carry significant practical drawbacks. Post-annealing contact morphology is characteristically rough, with pronounced grain structure that complicates photolithographic processing of contacts adjacent to fine-pitch gates. Process variability of 2–5× in $\rho_{c}$ from small differences in surface preparation, metal layer thickness, annealing ramp rate, and ambient is routinely observed, requiring tight in-line statistical process control. Crucially, the 800–900 °C annealing temperatures are thermally incompatible with gate dielectrics, passivation layers, or multilayer metal gate stacks already present on the device, constraining ohmic contact formation to the very early stages of the process flow and precluding back-end-of-line integration approaches [37]. The contact morphology also degrades gradually at device operating temperatures above ~300 °C, limiting the thermal ceiling of alloyed-contact GaN devices to below that achievable with the GaN material itself.

The GaN regrowth advantage is quantitatively explained by the regime transition (Table 2). Conventional contacts ($N_{D}$ ~ 2 × 10^19^ cm^−3^) operate at $E_{00}$ ~ 36 meV, marginally above kT, placing them in the TFE regime. Regrown contacts ($N_{D}$ > 5 × 10^20^ cm^−3^) achieve $E_{00}$ ~ 120 meV ≫kT, firmly establishing FE transport. This regime shift, not merely incremental doping increase, drives the two-order-of-magnitude improvement.

### 4.3. Epitaxial Regrowth

The transition from alloyed to epitaxially regrown n^+^ GaN contacts represents the most significant single advance in WBG contact technology of the past decade, delivering a two-order-of-magnitude improvement in $\rho_{c}$ and fundamentally reshaping the on-resistance landscape of advanced GaN HEMTs [51]. In regrowth contact schemes, the AlGaN barrier in source and drain regions is first selectively recessed or removed by dry or digital etch, and heavily Si-doped n^+^ GaN ($N_{D}$ > 10^20^ cm^−3^) is selectively re-deposited by metal–organic chemical vapor deposition (MOCVD) or molecular beam epitaxy (MBE) before metallization with Ti/Au or W/Au. The n^+^ GaN layer provides a near-metallic semiconductor surface with negligible Schottky barrier height to common metals, directly contacts the 2DEG at the underlying AlGaN/GaN heterointerface, and enables a $\rho_{c}$ of 2–5 × 10^−8^ Ω·cm^2^—one to two orders of magnitude below the best alloyed contact results [51,52].

Epitaxial regrowth of heavily doped GaN fundamentally restructures the contact problem. It is critical to understand that the contact resistance is NOT determined at the regrown/base GaN interface but rather at the metal/regrown-GaN interface. The regrown n^+^/n homojunction presents only a minimal potential step ($\Delta \varphi \;\sim \;kT\cdot ln(N_{heavy}/N_{base})$ ≈ 60–120 meV) across a quasi-neutral or minimally depleted region with negligible resistance contribution.

The transformative improvement arises from three synergistic factors:

(1) Ultra-high doping achievable in regrowth ($N_{D}$ > 10^20^ cm^−3^ via Si incorporation during MBE/MOCVD [52,53,54]), exceeding the ~2 × 10^19^ cm^−3^ limit of implantation or bulk doping due to surface depletion and damage. This reduces the Schottky depletion width at the metal/n^+^-GaN interface from W ~ 30 nm (conventional) to W ~ 5 nm (regrowth).
(2) Abrupt doping profile with epitaxial control providing < 5 nm transition width, versus graded profiles from implantation. This maximizes effective surface doping seen by the metal contact.
(3) Pristine interface formation with in situ metal deposition on as-grown n^+^ GaN surface, minimizing contamination and interface states that plague ex situ processed conventional contacts.

The metal/n^+^-GaN barrier ($\varphi_{Bn}$ ~ 0.8–1.0 eV) remains comparable to conventional contacts, but the ultra-thin depletion width shifts transport from thermionic-field emission (TFE) to a pure field emission (FE) regime, with $\rho_{c}\;\propto \;exp(\varphi_{Bn}/\sqrt{N_{D}})$ scaling explaining the two-order-of-magnitude improvement from ~10^−6^ to ~10^−8^ Ω·cm^2^ [52,54].

An underappreciated benefit of n^+^ GaN regrown contacts on AlGaN/GaN HEMTs is the elimination of polarization barrier penalties. Conventional contacts to the AlGaN barrier layer face an effective barrier $\varphi_{Bn}$ ~ 1.2–1.5 eV (including ~0.3 eV polarization contribution from high-Al content), as listed in Table 3. Regrown contacts place the metal/semiconductor interface on GaN (x = 0), where polarization is intrinsic to bulk GaN only, eliminating the AlGaN surface polarization penalty. Combined with ultra-high n^+^ doping, this contributes to the regrowth contact advantage.

The physical explanation for this dramatic improvement is straightforward: with $N_{D}>$ 10^20^ cm^−3^ and a favorable GaN electron effective mass (~0.20 $m_{0}$), the depletion width at any realistic Schottky barrier is narrow enough that tunneling probability approaches unity. The contact is no longer limited by barrier height—it is limited by the thermal velocity at the GaN/metal interface, which for common metals gives $\rho_{c}$ in the 10^−8^ Ω·cm^2^ range. This analysis illustrates the general principle that regrowth—by providing a near-metallic semiconductor contact surface—effectively decouples the contact resistance from the Schottky barrier physics of the bulk semiconductor, and it is this decoupling that makes regrowth the highest-priority contact strategy for all UWBG materials where barrier heights are prohibitively large.

A critical consequence of regrowth contact success is the shift in the dominant GaN HEMT source resistance component. With $\rho_{c}$ ~ 3 × 10^−8^ Ω·cm^2^ at a contact length of 1 μm (roughly 0.03 Ω·mm), the 2DEG access resistance from a 1 μm source-gate gap ($R_{sh}$ = 400 Ω/sq.) contributes ~0.4 Ω·mm—more than ten times the contact resistance. The on-resistance bottleneck has shifted from contact to access region, and further improvements in device performance now require either source-gate spacing reduction (with corresponding electric field management challenges), 2DEG sheet resistance reduction through barrier and channel optimization, or field-plate-assisted access resistance reduction.

### 4.4. Physical Explanation for Regrowth Advantage

The dramatic $\rho_{c}$ improvement from regrowth (3 × 10^−8^ vs. 3 × 10^−6^ Ω·cm^2^ for conventional) requires explicit physical explanation, as the mechanism is fundamentally different from barrier height reduction strategies. A critical clarification is necessary: the regrowth approach does **not eliminate or significantly reduce the Schottky barrier height**, $\varphi_{Bn}$, at the metal/n^+^-GaN interface. Ti/Au contacts on heavily doped n^+^ GaN still exhibit $\varphi_{Bn}$ ~ 0.8–1.0 eV, comparable to conventional Ti/Au on moderately doped GaN [51,52]. The transformative improvement arises not from barrier reduction but from ultra-thin depletion width enabling field emission with near-unity tunneling transmission.

The Schottky barrier depletion width scales as $W\;=\;\sqrt{2\varepsilon_{s}(\varphi_{Bn}\;-\;V)/(qN_{D})}$, creating fundamentally different transport regimes for conventional versus regrown contacts. For conventional contacts with $N_{D}$ ~ 2 × 10^19^ cm^−3^ and $\varphi_{Bn}$ ~ 1.0 eV, the depletion width extends approximately 30 nm at zero bias. The characteristic tunneling energy, $E_{00}\;=\;(q\hbar /2)\sqrt{N_{D}/m^{*}\varepsilon_{s}}$, is approximately 36 meV, which corresponds to 1.4kT at 300 K, placing the contact in the thermionic-field emission (TFE) regime. Tunneling probability through this 30 nm triangular barrier is only T ~ 10^−3^ to 10^−2^, requiring significant thermal activation over the barrier top to achieve measurable current flow.

In contrast, regrown contacts with $N_{D}\gg$ 5 × 10^20^ cm^−3^ and comparable $\varphi_{Bn}$ ~ 0.9 eV exhibit a depletion width of only 5 nm at zero bias, representing a factor of six reduction. This ultra-high doping increases the characteristic tunneling energy to $E_{00}$ ~ 120 meV, corresponding to 4.6kT at 300 K and firmly establishing field emission (FE) as the dominant transport mechanism. The tunneling probability through the 5 nm barrier increases dramatically to T ~ 0.5–0.8, approaching unity and enabling efficient carrier transport without thermal activation. This factor of 60× improvement in tunneling transmission directly translates to the observed two-order-of-magnitude $\rho_{c}$ reduction.

The band structure at the metal/n^+^-GaN interface reveals why the barrier persists despite the contact’s excellent performance. Fermi level pinning continues to occur at the metal–semiconductor interface, maintaining $\varphi_{Bn}$ ~ 0.8–1.0 eV through the same metal-induced gap states and interface chemistry that govern conventional contacts. However, ultra-high $N_{D}$ fundamentally alters the electrostatics: the screening length, $\lambda_{D}\;=\;\sqrt{\varepsilon_{s}\;kT/q^{2}N_{D}}$, contracts to approximately 1.5 nm, while band bending occurs over the distance W ~ 5 nm. This creates an electric field at the interface of $F\;\sim \;\varphi_{Bn}/W$ ~ 1.8 MV/cm, far exceeding the field in conventional contacts (F ~ 0.33 MV/cm). The combination of high field and thin barrier shifts the dominant transport mechanism from thermal emission over the barrier to quantum mechanical tunneling through it.

The term “near-metallic”, as applied to heavily doped n^+^ GaN, requires careful definition to avoid misinterpretation. This designation refers to the high free carrier concentration (>5 × 10^20^ cm^−3^) approaching the degeneracy condition where the Fermi level enters the conduction band, and Fermi–Dirac statistics become necessary. Additional characteristics include short screening length ($\lambda_{D}$ ~ 1.5 nm, comparable to atomic dimensions), the low bulk resistivity of the n^+^ layer itself (ρ ~ 10^−3^ Ω·cm, approaching metal-like values), and carrier concentrations sufficient to screen applied fields over nanometer distances. Critically, these properties describe the semiconductor layer’s electrical behavior but do not eliminate the Schottky barrier that forms when metal contacts this degenerately doped semiconductor. What changes is not the barrier’s existence but rather the tunneling efficiency through it.

The Wentzel–Kramers–Brillouin (WKB) approximation for tunneling transmission through a triangular barrier with field $F\;=\;\varphi_{Bn}/W$ provides a quantitative prediction of the observed improvement. The transmission probability, $T\;\approx \;\exp[-4\sqrt{2m^{*}}\varphi_{Bn}^{3/2}/(3\hbar qF)]$, depends exponentially on the electric field strength. For conventional contacts with W = 30 nm and F ~ 0.33 MV/cm, this yields T ~ 0.01 (1% transmission), requiring substantial series resistance for carriers to cross the interface. For regrown contacts with W = 5 nm and F ~ 1.8 MV/cm, the transmission increases to T ~ 0.6 (60% transmission), effectively rendering the barrier transparent to carrier flow. This factor of 60× improvement in transmission probability quantitatively accounts for the two-order-of-magnitude $\rho_{c}$ reduction observed experimentally.

Understanding why the regrown/base interface contributes negligibly to total contact resistance requires recognizing its fundamentally different character from a Schottky barrier. The n^+^/n homojunction between the regrown layer ($N_{D}$ ~ 5 × 10^20^ cm^−3^) and base GaN ($N_{D}$ ~ 10^17^–10^18^ cm^−3^) is a graded doping junction rather than a metal–semiconductor interface. The built-in potential step is thermal-scale, $\Delta \varphi \;\sim \;(kT/q)\ln(N_{heavy}/N_{base})$ ~ 120 meV, comparable to room-temperature thermal energy and far smaller than the eV-scale Schottky barriers discussed above. No depletion barrier exists because the majority of carriers flow energetically downhill from the n^+^ to n regions. The crystallographically continuous epitaxial interface lacks the interface states and roughness that characterize metal–semiconductor junctions. The resistance contribution from this interface is purely ohmic spreading resistance,$\;R_{interface}\;\sim \;\rho_{base}\;\times \;(\mathrm{geometry})$ ~ 10^−9^ Ω·cm^2^, three orders of magnitude below the metal/n^+^-GaN interface resistance. The current path, therefore, consists of the metal contacting n^+^ GaN through a Schottky barrier ($\rho_{c}$ ~ 10^−8^ Ω·cm^2^), followed by ohmic transport through the n^+^ layer and into the base GaN ($\rho_{c}$ ~ 10^−9^ Ω·cm^2^). The metal/n^+^-GaN interface determines the contact resistivity, not the regrown/base interface.

Three synergistic factors combine to enable this transformative improvement, each individually unattainable through conventional processing. First, ultra-high doping exceeding 5 × 10^20^ cm^−3^ can only be achieved via in situ epitaxial growth using molecular beam epitaxy or metalorganic chemical vapor deposition. Ion implantation followed by activation annealing is fundamentally limited to approximately 2 × 10^19^ cm^−3^ due to implant-induced lattice damage and incomplete dopant activation even after high-temperature annealing. Second, abrupt doping profiles with transition widths below 5 nm exploit the atomic-layer control available in epitaxial techniques. Implanted dopant profiles are inherently graded over 20–50 nm by ion straggling and diffusion during activation annealing, limiting the maximum effective surface concentration. Third, pristine interface formation through in situ metal deposition on the as-grown n^+^ surface minimizes atmospheric contamination and native oxide formation that degrade conventional ex situ processed contacts exposed to ambient conditions between semiconductor processing and metallization.

Conventional approaches to contact improvement demonstrate why they achieve only incremental gains rather than order-of-magnitude breakthroughs. Surface treatment methods, including HF etching and plasma cleaning, reduce interface state density and remove native oxides, but these modifications do not alter the fundamental doping concentration in the semiconductor contact region. The resulting improvement is limited to approximately 2× reduction in $\rho_{c}$ through modest barrier height reduction. Higher annealing temperatures, for example, increasing from 900 °C to 950 °C for Ti/Al stacks, promote more complete interfacial reactions and improved metal–semiconductor intermixing, but they cannot overcome the fundamental $N_{D}$ ~ 2 × 10^19^ cm^−3^ limit imposed by implant activation efficiency and solid solubility. Such optimization typically yields 3× improvement at best. In contrast, epitaxial regrowth fundamentally changes the doping regime by an order of magnitude, shifting from TFE to FE transport and delivering 100× improvement through a paradigm change rather than incremental optimization.

The universality of the underlying physics suggests this paradigm is transferable across WBG and UWBG material systems. The mechanism—ultra-high $N_{D}$ leading to ultra-thin W, enabling FE regime transport with high tunneling transmission T—depends only on achieving sufficient doping concentration relative to the material’s characteristic energy scales, not on GaN-specific properties. Indeed, β-Ga_2_O_3_ has demonstrated the approach with Sn- or Si-doped epitaxial contact layers, achieving $N_{D}$ ~ 10^20^ cm^−3^ and $\rho_{c}$ ~ 8 × 10^−7^ Ω·cm^2^ [55,56], proving applicability to oxide semiconductors. AlGaN-channel devices are under active development using selective n^+^ GaN regrowth in source-drain regions [57]. The primary challenge for diamond power devices—pending identification of a viable shallow *n*-type donor—would be immediately addressable through this epitaxial regrowth strategy if such a dopant were discovered. The implementation challenges center on selective-area epitaxy without facet formation, interface contamination during growth interruption, and thermal budget compatibility with pre-existing device structures, but these are engineering obstacles rather than fundamental physics limitations.

### 4.5. p-Type Contacts and Vertical Devices

*p*-type ohmic contacts to *p*-GaN are required for normally off *p*-GaN gate HEMTs, where the *p*-GaN gate structure must be electrically connected to the gate metal with low contact resistance. The standard approach uses Ni/Au or Pd/Au stacks annealed at 500–600 °C in oxygen-containing atmospheres, forming NiO or PdO interfacial oxides that promote hole injection; $\rho_{c}$ values of 10^−4^ to 10^−5^ Ω·cm^2^ are achieved on *p*-GaN with acceptor concentrations above 10^18^ cm^−3^ [50,58]. While sufficient for current *p*-GaN gate applications, the roughly one-to-two-order-of-magnitude gap relative to *n*-type contacts reflects the fundamental asymmetry discussed in Section 2. Ion implantation for source/drain contact doping—well established in SiC—is hampered in GaN by the need for implant activation above ~1300 °C, at which temperature GaN surface decomposition occurs; several groups have developed surface capping strategies (SiO_2_ and AlN caps) to extend the practical activation temperature range, with $\rho_{c}$ improvements approaching the one order of magnitude reported in optimized implantation-assisted contacts [59]. Vertical GaN device contacts—to *n*-type drain substrates and *n*-type drift layers—follow chemistry analogous to lateral HEMT contact metallurgy but typically at lower current density requirements, making moderate $\rho_{c}\;$(~10^−6^ Ω·cm^2^) from alloyed contacts acceptable for current vertical device performance levels [48].

## 5. Gallium Oxide (Ga_2_O_3_)

### 5.1. Material Context and Unique Characteristics

β-Ga_2_O_3_ has attracted rapidly growing research attention since approximately 2015, emerging as one of the most compelling UWBG candidates for power electronics [60]. The combination of a bandgap of ~4.8 eV, a predicted critical electric field of 6–8 MV/cm (BFOM ~3200× silicon), and the availability of large-area single-crystal substrates grown by melt-based methods (edge-defined film-fed growth, Czochralski)—practically unavailable for other UWBG materials—distinguishes β-Ga_2_O_3_ among its UWBG peers [12,61]. Homoepitaxial device structures have demonstrated blocking voltages exceeding 8 kV in Schottky barrier diodes, validating device-level potential [62].

The monoclinic crystal structure of β-Ga_2_O_3_ (space group C2/m) gives rise to pronounced anisotropy in electron effective mass (0.12–0.34 m_0_, depending on crystallographic direction) and thermal conductivity (10–27 W/m·K, direction-dependent)—substantially lower than SiC (~490 W/m·K) or GaN (~230 W/m·K). The low thermal conductivity is a serious thermal management constraint for high-power device operation and is increasingly recognized as a limiting factor that must be addressed at the packaging and device architecture level. Controllable *n*-type doping using Si, Ge, or Sn spans 10^15^–10^20^ cm^−3^ by homoepitaxial growth and ion implantation [55,63].

The defining physical constraint of β-Ga_2_O_3_ is the complete absence of viable *p*-type doping: no shallow acceptor exists in this material system, as confirmed by extensive theoretical calculations and experimental searches [53]. This restricts device architectures entirely to unipolar designs, eliminates *p-n* junction-based edge termination and bipolar operation, and simplifies the contact engineering challenge to a single polarity (*n*-type)—though this simplification is partly offset by the greater difficulty of achieving low $\rho_{c}$ to a UWBG *n*-type semiconductor compared to SiC or GaN.

### 5.2. Schottky Barrier Physics on β-Ga_2_O_3_

The electron affinity of β-Ga_2_O_3_ is approximately 3.7–4.0 eV, placing the minimum achievable Schottky barrier height in the Schottky–Mott limit (for metals with work functions above ~4.0 eV) at approximately 0–0.3 eV—seemingly favorable for ohmic contact. However, Fermi-level pinning with S ≈ 0.5–0.7 shifts the observed barrier heights for most practical metals to 0.8–1.5 eV, approximately twice the effective barrier in optimized SiC contacts [19,54]. The pinning state density is highest on the (010) and (001) surface orientations most commonly used for device fabrication, with different pinning positions on different facets requiring orientation-specific contact optimization.

The practical consequence is that achieving $\rho_{c}$ ~ 10^−6^ Ω·cm^2^ through direct metallization requires near-surface $N_{D}$ above ~10^19^ cm^−3^—achievable only with heavily doped contact layers or high-dose implantation. On substrates with $N_{D}$ ~ 10^17^ cm^−3^, typical of lightly doped drift layers, even optimally annealed direct Ti contacts show $\rho_{c}$ ~ 10^−3^ to 10^−4^ Ω·cm^2^—three orders of magnitude above the SiC baseline. This analysis establishes the device architecture requirement for β-Ga_2_O_3_: contact regions must be doped significantly above the channel/drift layer, requiring either selective regrowth, localized implantation, or heteroepitaxial contact layers.

The moderate Fermi-level pinning in β-Ga_2_O_3_ (S ≈ 0.6) represents a significant advantage over other UWBG materials [19,64]. Unlike AlN, where the charge neutrality level sits deep in the gap, causing severe pinning, β-Ga_2_O_3_ benefits from (i) a charge neutrality level (CNL) position relatively close to the conduction band, allowing for partial tunability with metal work function and (ii) a self-passivating oxide surface that minimizes reactive interface state formation. Experimentally, barrier heights vary from $\varphi_{Bn}$ ≈ 0.8 eV (Ti) to 1.5 eV (Pt), demonstrating the preserved metal work function dependence [64]. This moderate pinning, combined with achievable high doping ($N_{D}$ > 10^20^ cm^−3^) via Si or Sn donors, positions β-Ga_2_O_3_ favorably for contact engineering compared to AlN, where pinning is near-complete.

The transition concentration in β-Ga_2_O_3_ ($N_{D.transition}$ ~ 3.4 × 10^18^ cm^−3^) is similar to GaN despite a larger effective mass due to comparable permittivity. Epitaxial contact layers with $N_{D}$ ~ 10^20^ cm^−3^ approach the FE regime ($E_{00}$ ~ 90 meV), explaining competitive performance ($\rho_{c}$ ~ 10^−6^ Ω·cm^2^) with SiC despite a 1.5 eV larger bandgap.

### 5.3. Metallization Approaches and Recent Advances

Titanium-based contacts (Ti/Au and Ti/Al/Au) are the most widely studied system for *n*-type β-Ga_2_O_3_ [56]. Post-deposition annealing at 400–600 °C in an inert atmosphere promotes oxygen extraction by reactive Ti, forming TiO_x_ and an oxygen-deficient, quasi-metallic near-surface Ga_2_O_3_:Ti layer that reduces the effective barrier. On substrates with $N_{D}$ > 10^18^ cm^−3^, optimized Ti-based contacts yield $\rho_{c}$ ~ 10^−5^ to 10^−6^ Ω·cm^2^. A critical challenge specific to β-Ga_2_O_3_ is surface re-oxidation: even trace oxygen at elevated temperature rapidly reforms the surface oxide, shifting $\rho_{c}$ adversely; stringent inert atmosphere control during annealing is, therefore, mandatory [61]. In-based contacts (Indium Tin Oxide (ITO) interlayers; In-rich alloys) exploit indium’s lower work function (~4.1 eV) to reduce the initial Schottky barrier, achieving $\rho_{c}\;$~ 5 × 10^−6^ Ω·cm^2^ on moderately doped substrates [65]. Atomic Layer Deposition (ALD) Al_2_O_3_ interlayers of 0.5–1.5 nm reduce Fermi-level pinning by passivating interface states, lowering effective barrier height by 0.2–0.4 eV and yielding $\rho_{c}$ reductions of approximately one order of magnitude on moderately doped substrates [66].

The most impactful recent advance is the use of heavily doped epitaxial contact layers: Sn- or Si-doped β-Ga_2_O_3_ regrown in source/drain areas by MBE with $N_{D}$ > 10^20^ cm^−3^, followed by Ti/Au metallization [55]. This strategy—directly analogous to the GaN regrowth paradigm—achieves $\rho_{c}\;$ ~ 8 × 10^−7^ Ω·cm^2^, competitive with optimized SiC *n*-type contacts despite the 1.5 eV larger bandgap, by shifting to the full-FE regime where the contact is limited by thermal velocity rather than barrier height. Ion implantation with Si or Sn followed by RTA in oxygen-controlled atmosphere provides a patterning-compatible alternative to selective regrowth, with $\rho_{c}$ values of 10^−6^ to 10^−5^ Ω·cm^2^ demonstrated [54,67]. The In_2_O_3_/Ga_2_O_3_ heterointerface—where a low-bandgap In_2_O_3_ contact layer is lattice-matched to β-Ga_2_O_3_—is an emerging alternative that provides a favorable band alignment for *n*-type contact without requiring the extreme doping levels of the homoepitaxial regrowth approach [65].

A critical consideration for β-Ga_2_O_3_ contacts not present in nitride semiconductors is the role of oxygen vacancy migration in long-term stability. The native *n*-type doping in as-grown material often derives substantially from $V_{O}$, which can migrate with $E_{a}$ ~ 2.3–2.7 eV—placing it in the thermally activated regime at device operating temperatures. This motivates the development of epitaxial contact layers with intentional Si or Sn doping (replacing mobile $V_{O}$ with substitutional dopants) for improved reliability, discussed in Section 8.2.

### 5.4. Device Architecture Considerations

In lateral β-Ga_2_O_3_ MOSFETs, the absence of a polarization-induced 2DEG means that access resistance is governed by bulk channel doping (~10^17^ cm^−3^) rather than a high-density 2DEG sheet, making access resistance the dominant $R_{on}$ component even for optimized contacts and driving source-gate spacing minimization as the primary device-level optimization target [68]. In vertical β-Ga_2_O_3_ transistors—fin-channel FETs, current aperture devices—the contact geometry more closely resembles SiC MOSFETs; the back-contact to the heavily doped n^+^ substrate is straightforward ($\rho_{c}$ ~ 10^−6^ Ω·cm^2^), while the surface contact to the lightly doped drift layer benefits most from the heavily doped contact layer approach [69]. The thermal management challenge from low β-Ga_2_O_3_ thermal conductivity interacts directly with contact design: high current density at small contact areas creates localized self-heating that degrades both contact and channel performance, motivating composite substrate approaches (β-Ga_2_O_3_ on diamond, on AlN) and contact geometry optimization to distribute current density [70].

## 6. UWBG Frontier Materials: AlN/AlGaN and Diamond

### 6.1. AlN and High-Al-Content AlGaN: Extreme Contact Challenge

Wurtzite AlN has a direct bandgap of 6.1 eV and predicted critical field exceeding 12 MV/cm, giving a theoretical BFOM approximately 40× that of GaN and 40,000× that of silicon [71]. AlGaN alloys with Al fraction x > 0.5 offer a continuously tunable bandgap from 4.5 eV (x = 0.5) to 6.1 eV (x = 1), with strong spontaneous and piezoelectric polarization enabling 2DEG formation at Al-rich AlGaN heterointerfaces without intentional doping—a potentially transformative advantage over β-Ga_2_O_3_ for device architectures [57,72]. The primary application driver is deep-UV optoelectronics, but AlGaN-channel HEMTs are attracting increasing attention for power switching, given inherently higher breakdown voltage relative to GaN-channel counterparts.

AlN exhibits near-complete Fermi-level pinning (S ≈ 0.15–0.3) among the most severe of any semiconductor, with barrier heights largely independent of metal work function. This extreme pinning originates from the confluence of three factors: high MIGS density ($D_{it}$ > 10^14^ cm^−2^ eV^−1^) due to poor dielectric screening, a charge neutrality level positioned deep in the gap (~1.6 eV above CBM), and reactive surface chemistry forming native Al_2_O_3_. Direct metallization on AlN, therefore, yields consistently high barriers ($\varphi_{Bn}$ ≈ 1.8–2.2 eV) regardless of metal choice, explaining the uniformly poor contact resistivities (~10^−5^–10^−4^ Ω·cm^2^) reported for V/Au, Ti/Au, and even low-work-function metals. This contrasts sharply with β-Ga_2_O_3_, where moderate pinning (S ≈ 0.6) allows barrier engineering through metal selection to achieve a $\varphi_{Bn}$ as low as 0.8 eV with carefully chosen contact stacks.

The contact engineering challenge in this system is the most severe of any material reviewed. For Al_0.7_Ga_0.3_N with $E_{g}$ ≈ 4.5 eV and electron affinity of ~2.1 eV, a barrier-free *n*-type contact in the Schottky–Mott picture would require a metal with work function below 2.1 eV—lower than any stable metal (the lowest practical values are ~3.5–3.7 eV for Cs and Ba) [9,18]. Fermi-level pinning is near-complete in this system (S ≈ 0.1–0.3), leaving minimum achievable barrier heights of 1.5–2.5 eV regardless of metal selection. Silicon doping—the standard *n*-type approach in GaN—transitions to DX center behavior (a deep, electrically inactive large-lattice-relaxation defect) above an Al fraction of ~0.6, rendering Si doping progressively ineffective for creating free electrons in the high-Al-content composition range [64]. Germanium doping avoids DX center formation and has achieved active concentrations above 10^19^ cm^−3^ in Al_0.7_Ga_0.3_N, representing a significant enabling advance [73]. Oxygen acts as an *n*-type donor in AlN with an ionization energy of ~80–150 meV, but this results in only partial room-temperature ionization for Al fractions above 0.8.

AlN faces a fundamental doping–regime mismatch. The transition criterion requires $N_{D}$ > 3.1 × 10^18^ cm^−3^, but DX center formation limits achievable Si doping to ~10^18^ cm^−3^. Contacts thus operate at $E_{00}$ ≈ kT (TE/TFE borderline), where $\rho_{c}\;\propto \;exp(\varphi_{Bn}/kT)$ exhibits full exponential barrier dependence. Even with Ge doping partially suppressing DX centers, reaching the $N_{D}$ > 10^19^ cm^−3^ required for FE transport remains a critical challenge.

The most viable contact strategy for AlGaN-channel devices is selective-area regrowth of n^+^ GaN in source and drain contact regions, decoupling the contact metallurgy from the extreme barrier physics of the AlGaN channel [57]. This approach has demonstrated $\rho_{c}$ ~10^−6^ Ω·cm^2^ in proof-of-concept AlGaN-channel HEMT structures and represents the clearest path to device-competitive contact performance. However, it introduces a GaN/AlGaN heterointerface resistance component and requires a selective regrowth step that adds process complexity. For pure AlN contacts, vanadium (V/Au) is the most studied metallization, achieving $\rho_{c}\;$~8 × 10^−5^ Ω·cm^2^ at $N_{D}$ ~10^18^ cm^−3^—three orders of magnitude above the SiC baseline and far from device-competitive [71]. Polarization-engineered structures using graded AlGaN compositions to form quasi-2DEG channels with locally high carrier density offer an architecture that may tolerate moderate contact resistance if access region sheet resistance is sufficiently low through the high-density polarization-induced carrier sheet [72]. Tunnel junction injection structures—where a heavily doped *p*-type GaN or InGaN tunnel junction below the contact provides efficient electron injection from a metal with a lower work function requirement—are under active investigation for the AlN device context [74].

### 6.2. Polarization Effects on AlGaN Contact Barriers

High-Al-content AlGaN (x > 0.5) exhibits the most extreme polarization effects among nitrides. For Al_0.7_Ga_0.3_N, total polarization $P_{total}$ ≈ −0.110 C/m^2^ (Ga-polar) creates bound surface charge $\sigma_{pol}$ ≈ −6.9 × 10^13^ e^−^/cm^2^. This polarization-induced charge modifies contact barrier formation through mechanisms distinct from conventional Schottky physics:

(1) Pre-existing band bending: Before metal deposition, the polarization charge creates an electric field, $E_{pol}$ ≈ −1.5 MV/cm, over the AlGaN thickness, bending the conduction band upward by $\Delta V\;\sim \;E_{pol}\;\times \;t_{AlGaN}$. For typical 20 nm barriers, this contributes ~0.3 V to the effective barrier height.
(2) Metal work function independence: The polarization contribution, $\Delta V_{pol}$, is determined by the semiconductor structure (composition, thickness, and polarity), not by the metal. Experimental studies confirm that varying the metal work function from 4.0 to 5.5 eV produces only ~0.1–0.2 eV barrier height variation in high-Al AlGaN, far weaker than the 1.5 eV range predicted by Schottky–Mott theory, indicating polarization and pinning dominate over work function.
(3) Non-parabolic barrier profile: The superposition of polarization field (constant) and depletion field (varies with x) creates a tilted barrier with more triangular character near the interface. While this can reduce effective tunneling distance in some cases, the increased total barrier height ($\varphi_{Bn,\mathrm{eff}}$ ~ 1.5–1.8 eV for x = 0.7) dominates, yielding poor contact performance.
(4) Polarity engineering: N-polar growth reverses the polarization sign, creating positive surface charge and downward band bending. This can reduce effective barriers by ~0.3–0.4 eV, explaining reports of improved N-polar AlGaN contact performance despite typically higher defect densities in N-polar material.

The electron affinity of Al_0.7_Ga_0.3_N (χ ~ 2.0 eV) is low, but the effective barrier at metal interfaces is dominated by polarization-induced surface potential (~0.3 eV contribution) plus Fermi-level pinning (~0.8–1.0 eV from MIGS), yielding total $\varphi_{Bn}$ ~ 1.4–1.6 eV nearly independent of metal choice. This represents a fundamental challenge for direct AlGaN contacts not addressable through metal engineering alone.

### 6.3. Diamond: Unique Physics and P-Type Focus

Diamond occupies a singular position among WBG/UWBG semiconductors: it possesses the most extreme material properties—bandgap 5.47 eV, critical field >10 MV/cm, thermal conductivity 2200 W/m·K (highest of any bulk material), electron mobility up to 4500 cm^2^/V·s and hole mobility up to 3800 cm^2^/V·s—yet the gap between theoretical performance and demonstrated device metrics is the widest of any material reviewed [75,76]. The barriers are partly technological (small chemical vapor deposition (CVD) substrate sizes; difficult patterning) but substantially fundamental: the absence of a shallow *n*-type donor makes room-temperature *n*-type diamond devices physically unachievable.

Boron is the sole shallow acceptor in diamond ($E_{A}$ ≈ 370 meV), providing metallic *p*-type conductivity only above ~3 × 10^20^ cm^−3^ (Mott metal–insulator transition) [76]. No shallow donor exists: phosphorus has an ionization energy of ~570 meV (deep donor), nitrogen forms levels ~1.7 eV below the conduction band, and no other candidate has achieved useful *n*-type conductivity under ambient conditions [77]. Diamond power device research is, therefore, confined entirely to *p*-channel and surface-conductance architectures.

The most technically distinctive aspect of diamond contact physics is surface transfer doping on hydrogen-terminated diamond (H-diamond) [78,79]. When the CVD-grown surface is terminated with C–H bonds (resulting from hydrogen plasma exposure or the native CVD growth atmosphere), the surface has a negative electron affinity (NEA) of approximately −0.5 eV. Atmospheric adsorbates—particularly dissolved ionic species in a thin aqueous surface layer—spontaneously accept electrons from the diamond valence band, leaving a 2D hole gas (2DHG) with sheet densities of 10^12^–10^13^ cm^−2^ in the subsurface 2–10 nm region. Hall mobilities of 50–200 cm^2^/V·s at room temperature support FET operation, and H-diamond FETs have demonstrated $f_{T}$ > 100 GHz—competitive with GaN HEMTs in RF performance [80].

Ohmic contacts to the H-diamond 2DHG use high-work-function metals (Pd, Au, and Pt; $\varphi_{m}$ > 5.0 eV) to align with or below the 2DHG energy level; $\rho_{c}$ values of 10^−5^ to 10^−4^ Ω·cm^2^ are typical, limited by moderate 2DHG mobility rather than barrier height [80,81]. The primary practical limitation is thermal fragility: the H-terminated surface conductance is irreversibly destroyed above ~450 °C in air, and the physisorbed adsorbate layer is sensitive to surface chemistry changes during processing. Encapsulation with thermodynamically stable molecular acceptor layers—V_2_O_5_, MoO_3_, and WO_3_—that maintain transfer doping without reliance on atmospheric adsorbates has extended 2DHG stability to ~400 °C and represents the current state of the art for high-temperature H-diamond contacts [82].

Ohmic contacts to bulk heavily boron-doped diamond use either TiC formation (Ti deposition followed by carbothermal reduction of diamond at 700–900 °C, forming a low-resistance TiC layer) or laser graphitization of the near-surface region (converting sp^3^ diamond to conducting sp^2^ graphite in a ~20–50 nm subsurface layer by pulsed laser exposure) [83,84]. The laser graphitization approach achieves $\rho_{c}$ ~ 10^−6^ Ω·cm^2^ on heavily doped diamond and is compatible with standard photolithographic patterning. *n*-type diamond contacts remain an unsolved fundamental problem; the identification of a shallow donor with an ionization energy below ~100 meV would transform diamond’s device potential, but no viable candidate has emerged from extensive theoretical and experimental searches over three decades [76,77].

The hydrogen-terminated diamond surface creates a two-dimensional hole gas (2DHG) through surface transfer doping, where atmospheric adsorbates (H_2_O, O_2_) extract electrons from the diamond valence band, accumulating compensating holes within λ ~ 2–5 nm of the surface at sheet density $n_{s}$ ~ 10^13^ cm^−2^ [78,79]. While bulk diamond exhibits exceptional hole mobility ($\mu_{p,bulk}$ ~ 3800 cm^2^/V·s at 300 K for high-purity samples [75,76]), the surface-confined 2DHG suffers dramatic mobility degradation to $\mu_{2DHG}$ ~ 50–200 cm^2^/V·s [80,85]. This 20–75× mobility reduction arises from multiple surface-specific scattering mechanisms absent in bulk transport:

(1) Surface roughness scattering: Atomic-scale roughness (Δ ~ 0.3–1.0 nm RMS) couples strongly to carriers confined within λ ~ 3 nm. The scattering rate scales as τ^−1^ ∝ (Δ/λ)^2^, yielding $\mu_{SR}$ ~ 200–400 cm^2^/V·s contribution.
(2) Remote charged impurity scattering: The essential adsorbate layer (required for transfer doping) contains charged species at d ~ 0.5–2 nm from the 2DHG, acting as unscreened Coulomb scatterers with density comparable to carrier concentration. Contribution: $\mu_{CI}$ ~ 100–300 cm^2^/V·s.
(3) Surface phonon scattering: Enhanced coupling to C-H stretch modes (ℏω ~ 360 meV) and interface phonons due to broken symmetry and 2D confinement. Contribution: $\mu_{ph}$ ~ 300–600 cm^2^/V·s with strong temperature dependence.
(4) Surface state scattering: Incomplete H-termination creates dangling bonds and defects. Quality-dependent contribution: $\mu_{SS}$ ~ 50–200 cm^2^/V·s.
(5) Grain boundary scattering: In polycrystalline CVD diamond, grain boundaries introduce potential barriers. Contribution: $\mu_{GB}$ ~ 50–150 cm^2^/V·s for typical grain sizes.

Applying Matthiessen’s rule ($\mu_{total}^{-1}\;=\;\Sigma \mu_{i}^{-1}$), these mechanisms combine to yield observed $\mu_{2DHG}$ ~ 100–150 cm^2^/V·s for high-quality single-crystal H-diamond, degrading to 50–80 cm^2^/V·s for polycrystalline material.

Critical implication for contact resistance: The low 2DHG mobility creates high sheet resistance: $R_{sheet}\;=\;1/(q\cdot n_{s}\cdot \mu_{2DHG})$ ~ 3–12 kΩ/sq. For typical device geometries with contact spacing of L ~ 1–10 μm, the access resistance, $R_{access}\;=\;R_{sheet}\;\times \;(L/W)$, dominates the total contact resistance, often exceeding the metal/2DHG interface resistance, $R_{c}$, by factors of 10–100×. The interface contact resistivity $\rho_{c}$ ~ 5 × 10^−5^ Ω·cm^2^ is relatively good (low barrier height $\varphi_{Bp}$ ~ 0.3–0.5 eV from transfer doping), but device-level performance is limited by series resistance in the 2DHG.This fundamental limitation explains why bulk heavily doped diamond contacts (B > 10^21^ cm^−3^) using TiC or laser graphitization achieve superior performance ($\rho_{c}$ ~ 10^−6^ Ω·cm^2^) [84,85]: though bulk mobility is also degraded by ionized impurity scattering (μ ~ 100 cm^2^/V·s), three-dimensional conduction eliminates surface scattering and reduces sheet resistance to $R_{sheet}$ ~ 100 Ω/sq. (factor of 30–100× improvement over 2DHG); see Table 4.

The H-diamond case study illustrates a broader principle: 2D carrier confinement at surfaces introduces scattering mechanisms absent in bulk transport, fundamentally limiting contact performance through series resistance rather than interface resistance. AlGaN/GaN HEMTs face similar challenges—the 2DEG has μ ~ 1500–2000 cm^2^/V·s (vs. bulk GaN μ ~ 1000 cm^2^/V·s), but transfer length, $L_{T}$, is longer due to interface roughness and remote impurity scattering. However, the HEMT 2DEG benefits from:

- Light electron mass (vs. heavy holes in diamond);
- Larger carrier density ($n_{s}$ ~ 10^13^ cm^−2^ vs. 10^13^ cm^−2^ for H-diamond, similar);
- Remote scatterers farther away (20 nm AlGaN barrier vs. 1 nm adsorbate layer).

The diamond 2DHG represents the most extreme case of surface scattering limitations among WBG semiconductors.

### 6.4. Fundamental Limits of H-Diamond 2DHG Contacts

Even with perfect surface preparation (Δ → 0, eliminating roughness), the H-diamond 2DHG faces a fundamental mobility ceiling of μ ~ 300–500 cm^2^/V·s due to:

- Remote charged impurity scattering from the essential adsorbate layer required for transfer doping;
- Enhanced surface phonon coupling from 2D confinement;
- Heavier valence band effective mass ($m_{hh}^{*}$ ~ 0.7 $m_{0}$) compared to conduction electrons.

This fundamental limit, combined with saturation sheet density, $n_{s,max}$ ~ 2 × 10^13^ cm^−2^, yields a minimum achievable sheet resistance of $R_{sheet,min}$ of 800 Ω/sq.

For comparison, GaN regrown contacts achieve $R_{sheet}$ ~ 50 Ω/sq.—16× better despite comparable or higher carrier density—because electrons in bulk n^+^ GaN experience only ionized impurity and phonon scattering, not surface-specific mechanisms.

The 2D confinement represents an intrinsic disadvantage for contact applications. Diamond power device development has, therefore, shifted focus toward bulk p^+^ contact strategies (TiC/carbide formation, laser graphitization), which, despite lower mobility (~100 cm^2^/V·s), benefit from 3D current distribution and acceptable sheet resistance (~100 Ω/sq.).

## 7. Comparative Analysis

The field emission model predicts $\rho_{c}\propto \exp\left(C\varphi_{Bn}/\sqrt{N_{D}}\right)$, where the minimum achievable $\varphi_{Bn}$—bounded by Fermi-level pinning—scales approximately as 0.3–0.4 × $E_{g}$ across the materials reviewed, and effective mass increases monotonically with $E_{g}$. A fundamental scaling of minimum achievable $\rho_{c}$ thus emerges: approximately one order of magnitude per 0.8–1.0 eV increase in bandgap, evaluated at fixed $N_{D}$ and under material-appropriate parameters [1,6,8]. This scaling is not a technological limitation addressable by process improvements—it is a physical constraint that shifts the performance floor and defines the realistic target for each material system.

Compilation of best-demonstrated $\rho_{c}$ values across all five materials and silicon confirms this semi-empirical scaling over nearly eight orders of magnitude, from <10^−8^ Ω·cm^2^ for silicon CMOS and GaN regrown contacts to ~10^−5^ Ω·cm^2^ for H-diamond and ~10^−4^ Ω·cm^2^ for direct AlN metallization. The scatter around the scaling trend—spanning one to two orders of magnitude at each bandgap—quantifies the process improvement potential available within each material system before fundamental limits are approached. Materials currently far above the scaling line (β-Ga_2_O_3_ with conventional Ti contacts, AlGaN with direct metallization) have significant headroom for improvement through process optimization; those near the line (optimized GaN regrowth, SiC Ni silicide) are approaching their physical limits and require architectural changes for further progress.

Two noteworthy deviations from the general scaling merit explicit discussion. First, GaN regrown contacts lie substantially below the scaling trend predicted by direct-metal contact physics—a result of the regrowth strategy decoupling contact resistance from the bulk barrier height constraint by providing a near-metallic n^+^ semiconductor surface with a negligible effective barrier. This demonstrates that paradigm-level strategy changes can shift contact performance well below the fundamental floor that applies to direct metallization. Second, diamond H-surface contacts lie above the scaling trend—a consequence of the 2DHG density (~10^13^ cm^−2^) and mobility (~100 cm^2^/V·s) being lower than in the bulk-doped equivalent, partly offsetting the benefit of the transfer doping mechanism.

**Figure 4:**
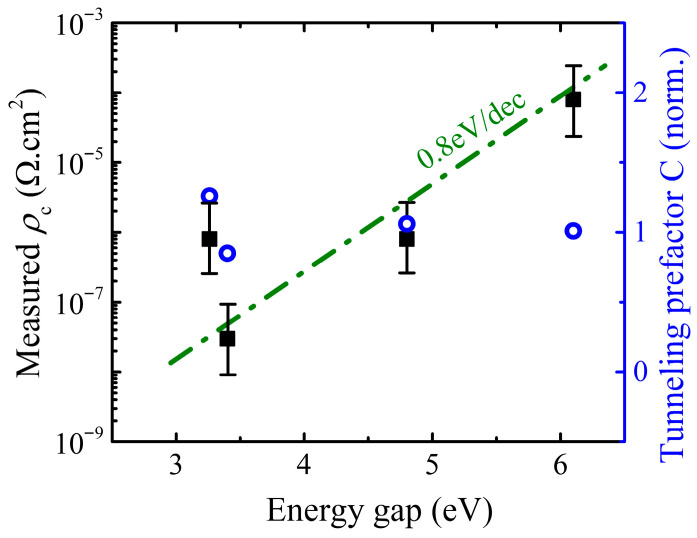
Cross-material scaling of minimum achieved specific contact resistivity with bandgap. The semi-empirical trend of ~1 order of magnitude per 0.8–1.0 eV increase reflects the combined effects of barrier height scaling ($\varphi_{Bn}\;\approx \;0.3-0.4\;\times \;E_{g}$), decreasing achievable doping, and increasing effective mass. Material-dependent variations in the tunneling prefactor C contribute ±20–30% logarithmic scatter around the trend. The black squares represent the experimental mean values of $\rho_{c}$ for various semiconductor materials, with vertical error bars indicating the measurement uncertainty or sample-to-sample variation. Blue open circles correspond to the calculated tunneling prefactor C (right axis) for each respective material.

The cross-material scaling is necessarily phenomenological rather than rigorously predictive, given the interplay of multiple material parameters, as illustrated in Figure 4. The constant C varies by ~2× from diamond (lowest $m^{*}$ and $\varepsilon_{s}$) to SiC (highest), contributing logarithmic scatter around the trend line. Additionally, Fermi-level pinning strength (quantified by parameter S) varies from near-complete pinning in AlN (S ≈ 0.15) to moderate pinning in GaN (S ≈ 0.3), causing $\varphi_{Bn}/E_{g}$ ratios to range from 0.25 to 0.40. Despite these variations, the empirical scaling holds to within ±0.5 orders of magnitude because (1) a barrier height increase with $E_{g}$ dominates over C variations when exponentially amplified, and (2) achievable $N_{D}$ systematically decreases with $E_{g}$ due to deeper donors and DX center formation in UWBG materials, acting synergistically with barrier effects. More details in Supplementary Materials Table S1.

The cross-material benchmark of *n*-type ohmic contact performance across WBG and UWBG semiconductors is summarized in Table 5.

**Figure 5:**
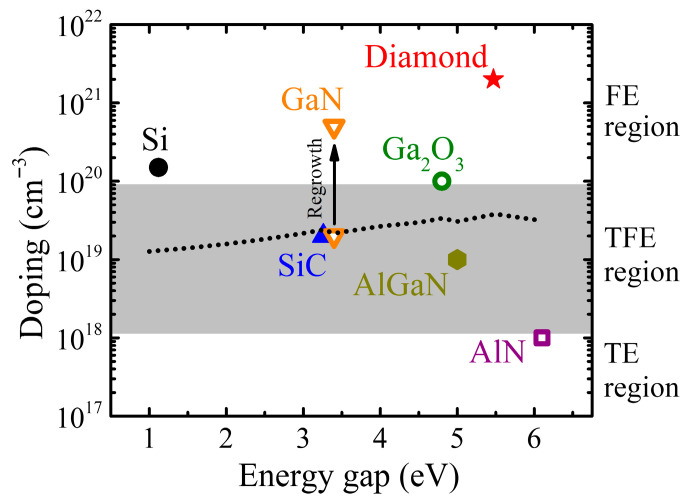
Transport regime classification map for WBG/UWBG contacts. Grey horizontal bands delineate the three transport regimes: field emission (FE, top), thermionic-field emission (TFE, middle), and thermionic emission (TE, bottom). The dotted line marks the TFE→FE transition boundary ($E_{00}=\;kT$ at 300 K). Materials in the FE regime achieve $\rho_{c}\;<$ 10^−6^ Ω·cm^2^, TFE regime materials achieve $\rho_{c}\;$ ~ 10^−6^-10^−7^ Ω·cm^2^, and TE regime materials exhibit $\rho_{c}\;>$ 10^−5^ Ω·cm^2^. The arrow shows GaN regrowth enabling transition from TFE to FE through ultra-high doping ($N_{D}$: 2 × 10^19^ → 5 × 10^20^ cm^−3^).

The benchmark table and scaling analysis yield several cross-cutting conclusions. The GaN regrown contact achieves the lowest $\rho_{c}$ of any WBG/UWBG material at device-relevant doping levels—lower even than optimized SiC implant-assisted contacts despite GaN’s larger bandgap—demonstrating that the achievable contact resistance is not determined by bandgap alone but by the product of effective barrier height, effective mass, and achievable surface doping and that regrowth is the most effective available strategy for optimizing all three simultaneously, see Figure 5. β-Ga_2_O_3_ achieves SiC-competitive $\rho_{c}$ when high-$N_{D}$ epitaxial contact layers are employed, positioning it as the most near-term viable UWBG material for practical low-$\rho_{c}$ applications. AlN direct metallization is three orders of magnitude worse than SiC at comparable doping and is unlikely to approach device-competitive performance without GaN regrowth or tunnel injection architectures. *p*-type contacts are consistently one to two orders of magnitude more resistive than *n*-type contacts where both are available, a material–physics asymmetry rather than a process immaturity that requires architectural responses (tunnel junctions; surface charge engineering) rather than metallization optimization alone.

The mechanistic uncertainty in SiC Ni contacts highlights a broader challenge in WBG contact physics: process-dependent interface structure variations obscure fundamental mechanisms. Unlike Si contacts where the Si/silicide interface is well characterized after decades of study, WBG metal/semiconductor interfaces remain incompletely understood. This gap impedes:

- Predictive modeling of new contact schemes;
- Transferring successful approaches between materials;
- Optimizing processing without extensive trial and error;
- Establishing physics-based reliability models.

Resolving these mechanistic questions requires advanced characterization (atomic-resolution STEM-EELS; operando measurements) combined with systematic process parameter studies and theoretical modeling. The GaN regrowth paradigm partly sidesteps this problem by replacing the poorly controlled metal/semiconductor interface with a well-defined epitaxial homojunction.

The GaN regrowth paradigm is often misunderstood as providing a “better interface” at the regrown/base junction. In reality, the regrown layer restructures the entire contact architecture by interposing an ultra-heavily doped epitaxial layer between the metal and the functional device layer, see Figure 5. The regrown/base interface itself is quasi-neutral and contributes negligible resistance. This architecture is directly transferable to other materials:

- β-Ga_2_O_3_: Homoepitaxial n^+^ layers ($N_{D}$ ~ 10^20^ cm^−3^ via Si doping) have demonstrated $\rho_{c}$ ~ 8 × 10^−7^ Ω·cm^2^ [74,80], proving the concept though not yet matching GaN performance.
- AlGaN: Regrown n^+^ GaN on AlGaN (rather than direct AlGaN contact) reduces $\rho_{c}$ by 2–3 orders.
- AlN: Requires Ge-doped GaN regrowth to avoid DX centers; direct AlN regrowth remains challenging.

The universal applicability stems from the physics: any semiconductor where $N_{D}$ > 10^20^ cm^−3^ can be achieved epitaxially will benefit from this architecture.

Polarization effects in wurtzite nitrides represent a unique contact physics phenomenon absent in centrosymmetric semiconductors (Si, SiC, diamond, and β-Ga_2_O_3_). The built-in electric fields and interface charges from spontaneous and piezoelectric polarization modify contact barriers by 0.2–0.4 eV independently of metal work function—making metal selection a secondary consideration compared to composition and polarity engineering. This explains the limited success of work function engineering in high-Al AlGaN contacts and motivates alternative approaches:

- Polarity inversion: N-polar growth to reverse polarization sign;
- Graded compositions: Spreading $\sigma_{pol}$ over distance to reduce field magnitude;
- Regrowth strategies: Contacting GaN rather than high-Al AlGaN;
- Tunnel junctions: Exploiting polarization-engineered band offsets.

The polarization contribution to barriers scales approximately linearly with Al composition ($\Delta V_{pol}$ ~ 0.4x eV), creating an additional challenge beyond bandgap widening for UWBG AlGaN contacts.

The oxygen vacancy migration issue in β-Ga_2_O_3_ illustrates a broader materials challenge: WBG/UWBG semiconductors with ionic bonding or oxide chemistry may exhibit defect migration mechanisms absent in covalent semiconductors. While GaN contacts show degradation primarily from re-oxidation at the metal interface or metallurgical aging (interdiffusion; phase transformations), β-Ga_2_O_3_ faces additional drift-driven dopant redistribution. This necessitates contact strategies specifically designed to stabilize or replace mobile defect species—an engineering consideration that becomes more prominent as materials diversity expands beyond traditional semiconductors, as illustrated in Table 6.

## 8. Device Context and Contact Engineering Strategies

### 8.1. Contact Resistance in the Device On-Resistance Budget

The device-level significance of contact resistance improvement is highly context-dependent, determined by the fraction of total, $R_{on}$, attributable to the contact at a given voltage rating and device architecture. The total on-resistance is $R_{on}\;=\;\;R_{contact}+\;R_{access}+\;R_{channel}+\;R_{drift}+\;R_{substrate}$. The theoretical minimum drift resistance scales as $R_{drift,min}\;=\;4V_{B}^{2}/(\varepsilon_{s}{\;\mu }_{n}\;E_{c}^{3})$, proportional to the inverse BFOM. At 1200 V, $R_{drift,min}$ is ~0.4 mΩ·cm^2^ for 4H-SiC, ~0.15 mΩ·cm^2^ for GaN, and ~0.05 mΩ·cm^2^ for β-Ga_2_O_3_ [1,86]. Contact resistance with $\rho_{c}$ ~ 10^−6^ Ω·cm^2^ and 1 μm contact length contributes approximately 0.1 mΩ·cm^2^—comparable to or exceeding $R_{drift,min}$ for these materials, confirming that contact resistance is a first-order device concern in this voltage range.

At lower blocking voltages, the contact significance intensifies sharply. For a 100 V GaN HEMT with $R_{drift}$ < 0.01 mΩ·cm^2^, alloyed contacts with $\rho_{c}$ ~ 5 × 10^−6^ Ω·cm^2^ contribute ~0.5 mΩ·cm^2^—more than 50 times the drift limit—completely negating the material’s theoretical advantage. This analysis explains the particular urgency of regrown contact development for sub-200 V GaN applications and generalizes to any WBG/UWBG material considered for low-voltage, high-frequency switching [4,47]. Conversely, above ~3300 V for SiC vertical devices where $R_{drift}$ dominates by design, contact quality is a secondary concern relative to drift layer purity and channel mobility, and the engineering priority shifts to contact reliability under sustained high-voltage stress.

In lateral GaN HEMTs with regrown contacts, the shift in dominant resistance from contact to 2DEG access region has changed device engineering priorities: further $\rho_{c}\;$ reduction below ~3 × 10^−8^ Ω·cm^2^ yields diminishing returns without simultaneous reduction of source-gate spacing or improvement of 2DEG sheet resistance. This transition—from contact-limited to access-limited—is a recurring theme as contact technology matures in each material system and signals that device architecture optimization must accompany contact engineering to translate materials advances into device performance gains [51]. In vertical SiC MOSFETs, the inversion channel resistance—limited by SiO_2_/SiC interface mobility of 20–50 cm^2^/V·s—is the largest single $R_{on}$ contributor at 1200 V, with $R_{contact}$ typically the second or third largest, followed by $R_{drift}$ [3].

Packaging interface resistance—die-attach solder or sintered-silver layer resistance (~0.5–2 mΩ·cm^2^) and wire-bond resistance (~0.1–0.5 mΩ·cm^2^)—contributes a fraction of total module resistance comparable to device contact resistance in high-performance WBG power modules and is frequently neglected in device-level analyses [87,88]. As device $\rho_{c}$ is reduced through advanced contact engineering, packaging interfaces increasingly constitute the dominant parasitic resistance at the module level, requiring co-optimization within an integrated module resistance budget.

### 8.2. Reliability Under High Stress

Contact reliability under sustained high-temperature, high-field, and high-current operation is as critical as initial $\rho_{c}$ for practical device deployment. In 4H-SiC, Ni_2_Si *n*-type contacts are stable to ~500 °C with acceptable morphological evolution; *p*-type Al-containing contacts degrade more rapidly above ~400 °C due to Al oxidation and outdiffusion, limiting their practical high-temperature ceiling [38,44]. In GaN HEMTs, current collapse—a transient increase in $R_{on}$ under high-voltage bias stress—has a partially contact-related origin: charge trapping near the contact edges and in the access region contributes to drain current degradation, and optimized contact geometry with proper surface passivation reduces but does not eliminate this effect [89]. In β-Ga_2_O_3_, oxygen vacancy migration under high electric field is a unique reliability concern: field-driven redistribution of oxygen vacancies (which act as donors) can dynamically alter the near-contact doping profile and shift $\rho_{c}$ by factors of 2–5 over a device’s lifetime, particularly at temperatures above 200 °C [67]. Diamond H-surface contacts are limited to <450 °C operating temperature by the thermal fragility of the hydrogen-terminated surface, placing an absolute ceiling on H-FET applications that higher-temperature molecular acceptor encapsulation partially addresses [82]. Contact electromigration at high current density—well characterized in silicon metallurgy—requires recalibration for the higher current densities and temperatures of WBG power contacts, and is an emerging area where material-specific electromigration coefficients and design rules are still being established.

Oxygen vacancy migration represents a unique reliability challenge in β-Ga_2_O_3_ contacts, distinct from mechanisms in nitride or carbide semiconductors. Oxygen vacancies ($V_{O}$) serve dual roles as the primary *n*-type dopant (shallow double donor, $E_{d}$ ~ 0.7 eV) and as mobile defect species. Unlike N vacancies in GaN or C vacancies in SiC, which are essentially immobile at typical operating temperatures, oxygen vacancies in the oxide lattice migrate with activation energy $E_{a}$ ~ 2.3–2.7 eV [63,77].

Under combined thermal and electric field stress at contact regions, the effective migration barrier reduces to $E_{a,\mathrm{eff}}$ ~ 1.9–2.5 eV due to field-assisted hopping (barrier reduction ΔE ~ qλF where λ ~ 2–3 Å is the hopping distance, and F is the local field). At operating temperatures of 150–200 °C with contact edge fields of 1–3 MV/cm, $V_{O}$ migration becomes significant with characteristic time constants of hours to days.

The near-contact region experiences maximum degradation due to: (i) electric field concentration from current crowding driving $V_{O}$ drift, (ii) thermal hotspots from $I^{2}R_{c}$ heating accelerating migration exponentially, (iii) chemical potential gradients at the metal/semiconductor interface, and (iv) self-amplifying effect where $V_{O}$ depletion reduces local $N_{D}$, increasing depletion width and field further. This creates temporal evolution of the doping profile $N_{D}\left(x,t\right)$ governed by drift–diffusion–reaction dynamics:

$$\partial N_{V}/\partial t\;=\;D_{V}\cdot \partial^{2}N_{V}/\partial x^{2}\;-\;\mu_{V}\cdot F\cdot \partial N_{V}/\partial x\;-\;k_{ox}\cdot N_{V}$$

where $D_{V}$ ~ 10^−14^ cm^2^/s at 200 °C is the vacancy diffusion coefficient, $\mu_{V}$ is the field-driven mobility, and $k_{ox}$ is the re-oxidation rate at interfaces.

Observed degradation mechanisms include:

(1) $V_{O}$ depletion: Drift away from cathode contacts reduces local $N_{D}$ from ~10^20^ to ~10^19^ cm^−3^, increasing $\rho_{c}$ by factors of 3–5× over 100–1000 h at 200 °C.
(2) Re-oxidation barrier formation: $V_{O}$ accumulation at the metal interface followed by oxygen uptake forms insulating layers, manifesting as increased series resistance or turn-on voltage in diodes [88].
(3) Spatial inhomogeneity: Preferential $V_{O}$ migration along grain boundaries creates conducting filaments or depleted regions, increasing contact noise and $\rho_{c}$ fluctuation.

Accelerated aging studies at multiple temperatures yield degradation activation energy $E_{a}$ ~ 2.0–2.4 eV, consistent with $V_{O}$ migration as the primary mechanism. Extrapolated device lifetime at 150 °C operation reaches 10^4^–10^5^ h (acceptable for many applications) but exhibits strong temperature sensitivity with ~10× reduction per +50 °C.

Mitigation strategies under development include: (i) oxygen-rich annealing and ALD encapsulation suppressing $V_{O}$ formation/out-diffusion, (ii) epitaxial contact layers with intentional Si-doping replacing $V_{O}$ as donor source (demonstrating improved stability [64,80]), (iii) thin Al_2_O_3_ interlayers blocking $V_{O}$/metal interaction, and (iv) field management through edge termination reducing driving force for migration.

### 8.3. Temperature Scaling of Contact Resistivity

The temperature dependence of specific contact resistivity $\rho_{c}$(T) is governed by the operative transport mechanism, which itself may shift with temperature. The three regimes exhibit distinct thermal signatures:

Field emission (FE): $\rho_{c}$ ∝ exp($C\varphi_{Bn}/\sqrt{N_{D}}$) with weak temperature dependence from $C(T)\;\propto \;\sqrt{m^{*}(T)\varepsilon_{s}(T)}$. Both $m^{*}$ and $\varepsilon_{s}$ vary by <10% over 25–500 °C, yielding typical d$\rho_{c}$/dT ~ 0.1–0.3%/K—nearly temperature-independent behavior.

Thermionic-field emission (TFE): $\rho_{c}\propto \;\exp[\varphi_{Bn}/(E_{00}\coth(E_{00}/kT))]$ exhibits intermediate temperature dependence. At T well below $E_{00}$/k, the coth term approaches unity and behavior resembles FE; at $T\;\gg \;E_{00}/k$, activation approaches TE character. Typical activation energies $E_{a,\mathrm{eff}}\;=\;\partial \ln(\rho_{c})/\partial (1/kT)$ range 0.1–0.4 eV depending on $E_{00}$.

Thermionic emission (TE): $\rho_{c}\;\propto \;\exp(q\varphi_{Bn}/kT)$ shows strong thermal activation with $E_{a}\;=\;q\varphi_{Bn}$. For WBG/UWBG materials with $\varphi_{Bn}$ = 0.8–2.0 eV, this yields dramatic $\rho_{c}$ reduction: factors of 10–100× from 25 to 500 °C.

Material-specific temperature behavior to 500 °C (see Table 7):

Si (FE regime at all T): $\rho_{c}$ decreases by <2× from 25 to 500 °C. High $N_{D}$ (>10^20^ cm^−3^) maintains FE transport and minimal thermal activation. Metallurgical stability (silicide phase transformations) limits practical operation above ~400 °C for most contacts.

4H-SiC *n*-type (TFE regime): Ni_2_Si contacts exhibit $\rho_{c}$ reduction of 3–5× from 25 to 500 °C with activation energy $E_{a}$ ~ 0.15–0.25 eV [33,38]. The modest activation reflects TFE character ($E_{00}$ ~ 55 meV at $N_{D}$ ~ 2 × 10^19^ cm^−3^). Morphological stability to ~500 °C enables reliable high-temperature operation. *p*-type Al-containing contacts show larger activation ($E_{a}$ ~ 0.3–0.5 eV) but degrade above ~400 °C due to Al oxidation [38,44].

GaN conventional (TFE regime): Ti/Al alloyed contacts show $\rho_{c}$ reduction of 5–10× from 25 to 500 °C ($E_{a}$ ~ 0.2–0.4 eV) as thermal energy enhances TFE probability. However, re-oxidation of the Ti/Al interface above ~400 °C can cause time-dependent degradation, limiting sustained high-T operation [37].

GaN regrown (FE regime): Ultra-high doping ($N_{D}$ > 5 × 10^20^ cm^−3^) yields $E_{00}$ ~ 120 meV ≫ kT even at 500 °C (kT ~ 67 meV). Contacts exhibit nearly temperature-independent $\rho_{c}$ (~30% decrease over 25–500 °C), confirming FE transport dominance. This weak temperature dependence is a key reliability advantage [51,52].

β-Ga_2_O_3_ (TFE/FE boundary): Epitaxial contact layers ($N_{D}$ ~ 10^20^ cm^−3^) operate near the regime transition. At 300 K, $E_{00}$ ~ 90 meV ≈ 3.5 kT favors TFE. At 500 °C (kT ~ 67 meV), $E_{00}/kT$ increases to ~1.3, strengthening FE character. Observed $\rho_{c}$ reduction is modest (2–4×) over 25–500 °C. The dominant reliability concern is oxygen vacancy migration rather than contact mechanism transition [67,77].

AlN (TE/TFE regime): DX-center-limited doping ($N_{D}$ ~ 10^18^ cm^−3^) yields $E_{00}$ ~ 26 meV ≈ kT at 300 K—deep in TE/TFE crossover. Direct V/Au contacts exhibit strong thermal activation with $E_{a}$ ~ 0.6–1.0 eV, producing $\rho_{c}$ reduction of 10–50× from 25 to 500 °C [71]. This large activation reflects the high barrier ($\varphi_{Bn}$ ~ 1.8–2.2 eV) and borderline TE character. However, practical benefit is limited by the absolute $\rho_{c}$ magnitude (~10^−5^ Ω·cm^2^ even at 500 °C).

Diamond H-surface (thermal ceiling at ~450 °C): The hydrogen-terminated surface undergoes irreversible degradation above 400–450 °C in air due to H desorption and surface reconstruction, eliminating transfer doping [82]. Below this threshold, $\rho_{c}$ shows weak temperature dependence (~2× decrease over 300–400 °C) characteristic of the already-high 2DHG sheet density. V_2_O_5_-stabilized contacts extend operation to ~400 °C but cannot reach 500 °C [82].

Transport mechanism transitions: For materials operating in a TFE regime at room temperature (SiC, conventional GaN, or Ga_2_O_3_), elevated temperature produces a shift toward TE character as kT becomes larger relative to $E_{00}$. This counterintuitive trend—where thermal energy enables surmounting barriers classically rather than tunneling—occurs because $E_{00}$ is temperature-independent while kT increases. However, for device-relevant doping levels ($N_{D}$ > 10^19^ cm^−3^), the transition is gradual rather than abrupt, and most contacts remain in the TFE regime even at 500 °C.

### 8.4. Advanced Contact Engineering Strategies

Epitaxial regrowth is the highest-impact strategy identified in this review, applicable to GaN (established), β-Ga_2_O_3_ (recently demonstrated), and high-Al-content AlGaN (in development). By providing a near-metallic n^+^ semiconductor surface with a negligible barrier to common metals, regrowth decouples contact resistance from the bulk Schottky barrier physics and enables the two-order-of-magnitude $\rho_{c}$ improvement demonstrated in GaN [51,55]. Process integration challenges include selective-area growth without facet formation or interface contamination, and thermal budget management to avoid degradation of underlying channel structures. The near-term priority is reliable, reproducible regrowth contact integration in β-Ga_2_O_3_ and AlGaN device process flows, following the trajectory established by GaN technology.

ALD dielectric interlayers of 0.5–1.5 nm thickness (Al_2_O_3_, HfO_2_, ZnO) reduce Fermi-level pinning by passivating interface states, lowering the effective barrier height by 0.2–0.4 eV and yielding $\rho_{c}$ reductions of up to one order of magnitude across GaN, β-Ga_2_O_3_, and AlN systems [66,90,91]. The mechanism requires sufficiently thin interlayers that direct tunneling resistance is negligible. Degradation under bias stress—charge injection into the dielectric that degrades passivation—is the primary reliability concern. Surface dipole engineering through alkali–halide monolayers (CsF) or self-assembled molecular monolayers provides analogous barrier height reduction of 0.3–0.5 eV with high chemical tunability, though thermal stability below ~400 °C limits compatibility with high-temperature contact annealing in SiC and GaN [92,93]. Alternative *n*-type dopants—particularly Ge in AlGaN, which avoids DX center formation and enables $N_{D}$ > 10^19^ cm^−3^ in Al-rich compositions—address the doping bottleneck for contact regions in UWBG materials [73].

AI/ML-guided process optimization is demonstrating efficiency gains of 3–5× in parameter-space exploration for contact metallization in GaN and β-Ga_2_O_3_ [94,95]. Bayesian experimental design with physics-informed surrogate models reduces the number of experiments required to find optimal annealing temperature, metal stack composition, and surface preparation sequences—particularly valuable in UWBG systems where each experiment requires costly substrate material and extensive characterization. 2D material interlayers—graphene (work function tunable over ~1 eV range through doping) and MXenes (metallic conductivity; chemical compatibility with III-nitride surfaces)—are under early-stage investigation as contact mediators [29,96,97,98]. Integration challenges (layer quality at small area, lateral conductance shorting between adjacent contacts, and thermal stability) and the absence of systematic material-specific optimization data currently limit 2D interlayers to proof-of-concept demonstrations, but the physical concept is sound and warrants continued investigation as processing control improves. Ferroelectric interlayer contacts—exploiting the negative capacitance effect in ferroelectric HfO_2_ or BaTiO_3_ interlayers to provide effective barrier height amplification—represent an emerging theoretical concept with limited experimental validation in WBG systems but potential for barrier reduction in the most challenging UWBG contact configurations [85,99].

## 9. Conclusions and Outlook

This review has examined contact resistance phenomena across five WBG and UWBG semiconductor systems—4H-SiC, GaN, β-Ga_2_O_3_, AlN/AlGaN, and diamond—from fundamental physics through characterization methodology, material-specific state of the art, device context, and advanced engineering strategies.

The central quantitative finding is a fundamental exponential scaling of minimum achievable specific contact resistivity with the bandgap: an approximately one order of magnitude increase in $\rho_{c}$ per 0.8–1.0 eV increase in $E_{g}$, arising from the interplay of increasing minimum Schottky barrier height (through Fermi-level pinning), increasing carrier effective mass, and decreasing achievable near-surface doping concentration. This is not a technological limitation correctable by process optimization—it is a physical constraint that defines the realistic performance floor for each material system. Process engineering can shift each material’s contact performance toward this floor but cannot systematically transcend it without paradigm-level changes in contact architecture.

The GaN transition from alloyed ($\rho_{c}$ ~ 5 × 10^−6^ Ω·cm^2^) to epitaxially regrown ($\rho_{c}$ ~ 3 × 10^−8^ Ω·cm^2^) contacts is the clearest demonstration that paradigm-level architectural change is achievable, delivering two orders of magnitude improvement and fundamentally shifting the GaN HEMT on-resistance bottleneck from contact to access region. Epitaxial regrowth is identified as the highest-priority contact engineering research direction for β-Ga_2_O_3_ and AlGaN systems, with recent demonstrations in β-Ga_2_O_3_ establishing feasibility and confirming the strategy’s transferability across the WBG/UWBG material family.

Among the five material systems, the principal status conclusions are as follows. 4H-SiC *n*-type contacts are approaching the fundamental limit imposed by nitrogen solubility, with incremental improvement possible through implant optimization but no order-of-magnitude gains available without new contact strategies; *p*-type contacts remain the primary unresolved SiC contact challenge. GaN regrown contacts have effectively solved the *n*-type contact problem for current device applications, with further performance gains requiring access resistance and 2DEG architecture improvement. β-Ga_2_O_3_ is best positioned among UWBG materials, with epitaxial-layer contacts achieving SiC-competitive $\rho_{c}$ and a clear development roadmap toward practical device integration. AlN/high-Al AlGaN presents the most severe contact challenge—with direct metallization three orders of magnitude above SiC baseline—and will require GaN regrowth, Ge doping, and tunnel junction approaches in combination. Diamond *p*-type and H-surface contacts are functional at the level required for high-frequency device demonstration, while *n*-type diamond contacts await the fundamental materials science discovery of a viable shallow donor.

The rapid evolution of WBG and UWBG contact technology is reflected in recent comprehensive reviews addressing β-Ga_2_O_3_ contact physics and device applications [100,101]; cross-material perspectives on metal–semiconductor interfaces spanning Ga_2_O_3_, SiC, diamond, and other emerging semiconductors [102]; the broader transition from wide to ultrawide-bandgap devices for power and RF electronics [103]; and experimental demonstration of high-temperature contact stability in AlGaN/GaN heterostructures to 500 °C [104]. These material-specific and thematic analyses complement the cross-material comparative framework developed in this review and collectively document the field’s progression from empirical metallization optimization toward physics-based contact engineering grounded in transport regime identification and epitaxial regrowth strategies.

Looking forward, five research priorities stand out as most likely to determine the pace of progress in WBG and UWBG contact technology over the next decade. First, reliable regrown contact integration in β-Ga_2_O_3_ and AlGaN-channel device process flows—achieving reproducible $\rho_{c}$ < 10^−6^ Ω·cm^2^ across wafer-scale substrates—is the most immediately impactful materials engineering challenge. Second, the identification of a shallow donor for *n*-type diamond would be a transformative materials discovery, unlocking the full device potential of the material with the most extreme physical properties of any in this review. Third, community adoption of standardized $\rho_{c}$ measurement and reporting protocols—specifying cleaning chemistry, TLM geometry rationale, bias linearity verification, and temperature-dependent supplementary measurements—would dramatically reduce the current one-to-two-order-of-magnitude inter-laboratory scatter that impedes genuine technology assessment. Fourth, module-level co-optimization of device contact resistance and packaging interface resistance is increasingly necessary as WBG device contacts are optimized to the point where packaging contributes comparably to total module resistance. Fifth, systematic application of AI/ML-guided experimental design to UWBG contact metallization optimization—where the parameter space is vast, experiments are costly, and the available literature dataset remains sparse—offers a disproportionate acceleration potential relative to purely empirical approaches and deserves to become a standard tool in the contact engineering research toolkit. Progress on these fronts, grounded in the physical framework established across all five material systems in this review, will determine whether the extraordinary material properties of UWBG semiconductors can be fully harnessed in the next generation of power electronic devices.

## Figures and Tables

**Table 1:** Effective mass impact on *p*-type vs. *n*-type contacts.

Material	$m_{e}^{*}$ ($m_{0}$)	$m_{hh}^{*}$($m_{0}$)	Mass Ratio	$\sqrt{Ratio}$	$T_{p}/T_{n}$ ^(a)^	Doping Ratio ^(b)^	Total $\rho_{c,p}/\rho_{c,n}$
Si	1.08	0.49	0.45	0.67	~2×	~0.5×	~1×
SiC	0.40	1.5	3.75	1.94	~0.1×	~0.2×	~50×
GaN	0.20	1.6	8.0	2.83	~0.05×	~0.1×	~200×
Ga_2_O_3_	0.28	4.0	14.3	3.78	~0.02×	~0.05×	~1000×
Diamond	0.20	0.7	3.5	1.87	~0.1×	>10× ^(c)^	~10×

^(a)^ Tunneling transmission ratio, T ∝ exp(−2κd), where $\kappa \;\propto \;\sqrt{m^{*}}$; ^(b)^ achievable *p*-type/*n*-type doping density ratio; ^(c)^ diamond exception: ultra-high B doping (>10^21^ cm^−3^) compensates for mass penalty.

**Table 3:** Polarization parameters for AlGaN.

Composition	$P_{SP}$ (C/cm^2^)	$P_{PE}$ (C/cm^2^)	$P_{total}$ (C/cm^2^)	$\sigma_{pol}$ (10^13^/cm^2^)	${∆V}_{pol}$(V)	$\varphi_{Bn,typical}$ (eV)
GaN	−0.034	0	−0.034	−2.1	~0	0.9
Al_0.3_Ga_0.7_N	−0.053	−0.018	−0.071	−4.4	0.15	1.1
Al_0.5_Ga_0.5_N	−0.067	−0.028	−0.095	−5.9	0.23	1.3
Al_0.7_Ga_0.3_N	−0.078	−0.035	−0.113	−7.1	0.31	1.5
AlN	−0.090	0	−0.090	−5.6	0.20	1.8

**Table 4:** Scattering mechanisms in H-diamond 2DHG vs. bulk.

Mechanism	Bulk Diamond	H-Diamond 2DHG	Ratio	Physical Origin
Acoustic phonon	3800	—	—	Dominant in bulk at RT
Surface roughness	—	200–400	—	Δ/λ ~ 0.15 coupling
Charged impurities	—	100–300	—	Adsorbate charge d ~ 1 nm
Surface phonons	—	300–600	—	C-H modes, 2D confinement
Surface states	—	50–200	—	Dangling bonds, defects
Total mobility	3800	50–200	20–75×	
Sheet resistance	~50 Ω/sq. *	3–12 kΩ/sq.	60–240×	

* Sheet resistance for bulk calculated from 1 μm thick p^+^ layer (p^+^ >10^21^ cm^−3^).

**Table 5:** Cross-material benchmark of *n*-type ohmic contact performance across WBG and UWBG semiconductors. BFOM = $\mu E_{c}^{3}$ relative to silicon; best $\rho_{c}$ values represent optimized research results, not process-average performance. S = Fermi-level pinning parameter.

Material	$E_{g}$ (eV)	$E_{c}$ (MV/cm)	BFOM (rel. Si)	$\mathrm{Best}\;\rho_{c}$ (Ω·cm^2^)	Strategy [Reference]	$N_{D}$ (cm^−3^)	S
Si	1.12	0.3	1	<10^−8^	NiSi_2_ [22]	>10^20^	~0.1
4H-SiC	3.26	2.5	~340	~8 × 10^−7^	Ni silicide + implant [32,40]	~2 × 10^19^	~0.3
GaN	3.4	3.3	~870	~3 × 10^−8^	n^+^ GaN regrowth/Ti:Au [51,52]	>10^20^	~0.3
β-Ga_2_O_3_	4.8	~7	~3200	~8 × 10^−8^	Epi contact layer/Ti:Au [55,56]	~10^20^	~0.6
Al_0.7_Ga_0.3_N	5.29	~8	~5000	~10^−6^	n^+^ GaN regrowth [57]	2DEG	~0.2
AlN	6.1	~12	~40,000	~8 × 10^−5^	V/Au direct [71]	~10^18^	~0.15
Diamond (p, bulk)	5.47	~10	~25,000	~10^−6^	TiC/laser graphit. [83,84]	B > 10^21^	—
Diamond (H-surf.)	5.47	~10	~25,000	~5 × 10^−5^	Pd/Au on 2DHG [80,81]	~10^13^ cm^−2^	—

**Table 6:** Dominant contact degradation mechanisms by material. $T_{50}$ represents time to 50% increase in $\rho_{c}$ under bias stress at 200 °C.

Material	Primary Mechanism	$E_{a}$ (eV)	$T_{50}$ at 200 °C	Mitigation
SiC	Ni/Al interdiffusion	1.5–1.8	>10^5^ h	Ta barrier layer
GaN	Re-oxidation at Ti/Al	1.2–1.5	>10^4^ h	Encapsulation
Ga_2_O_3_	$V_{O}$ migration	2.0–2.4	10^3^–10^4^ h	Si-doped epi layer
Diamond	H desorption (H-surf.)	2.8–3.2	<10^2^ h	V_2_O_5_ stabilization

**Table 7:** Temperature dependence of contact resistivity.

Material	Regime (RT)	$E_{a}$ (eV)	$\rho_{c}$$(500\;{}^{\circ}C)/\rho_{c}$(RT)	Limiting Factor
Si	FE	<0.5	~0.5×	Silicide stability
SiC (n-type)	TFE	0.15–0.25	~0.2–0.3×	Morphology stable
SiC (p-type)	TFE	0.3–0.5	~0.05–0.1×	Al oxidation > 400 °C
GaN (conv.)	TFE	0.2–0.4	~0.1–0.2×	Ti/Al re-oxidation
GaN (regrown)	FE	<0.1	~0.7×	Excellent stability
β-Ga_2_O_3_	TFE/FE	0.1–0.2	~0.3–0.5×	$V_{O}$ migration
AlN	TE/TFE	0.6–1.0	~0.02–0.1×	Still too high $\rho_{c}$
Diamond	—	—	Degrades	H desorption < 450 °C

## Data Availability

No new data were created or analyzed in this study. Data sharing is not applicable to this article.

## Supplementary Materials

The following supporting information can be downloaded at https://www.mdpi.com/article/10.3390/ma19071424/s1: Table S1: Cross-material scaling of minimum achieved specific contact resistivity with bandgap.
