# Supplementary material for: Ohmic Contact Resistance in Wide-Bandgap and Ultrawide-Bandgap Power Semiconductors: From Fundamental Physics to Interface Engineering
Source: Materials (Basel). 2026 Apr 2;19(7):1424. doi: 10.3390/ma19071424 (PMC13074107; doi:10.3390/ma19071424)
Supplement: Supplementary file 1 [file materials-19-01424-s001.zip › materials-4189066-supplementary.pdf]

materials

MDPI

Supplementary material

Ohmic Contact Resistance in Wide-Bandgap and Ultrawide-Bandgap Power Semiconductors: From Fundamental Physics to Interface Engineering

Martin Weis [1,*]

[1] Slovak University of Technology in Bratislava, Ilkovicova 3, 841 04 Bratislava, Slovakia; martin.weis@stuba.sk
* Correspondence: martin.weis@stuba.sk

Academic Editor: Firstname Lastname

Received: date
Revised: date
Accepted: date
Published: date

Citation: To be added by editorial staff during production.

Copyright: © 2025 by the authors. Submitted for possible open access publication under the terms and conditions of the Creative Commons Attribution (CC BY) license (https://creativecommons.org/licenses/by/4.0/).

S1. Mathematical Derivation of Semi-Empirical Scaling Law

S1.1 Field Emission Equation and Constant C

The field emission current density through a Schottky barrier is given by:

$$J = A^*T^2\exp(-q\varphi_{Bn}/kT)\ \exp(\sqrt{qE/E_{00}})$$

where $E_{00} = (q\hbar/2)\sqrt{N_D/m^*\varepsilon_s}$ is the characteristic tunneling energy.

In the heavy doping limit ($E_{00} \gg kT$), the specific contact resistivity becomes:

$$\rho_c = (\partial J/\partial V)^{-1}|_{(V=0)} \propto \exp(C\varphi_{Bn}/\sqrt{N_D})$$

where the constant C is:

$$C = (4\pi/h)\sqrt{2m^*\varepsilon_s} = (4\pi/h)\sqrt{2m^*\varepsilon_0\varepsilon_r}$$

Explicit evaluation:

h = 6.626×10^(-34) J·s
ε_0 = 8.854×10^(-12) F/m
m_0 = 9.109×10^(-31) kg
q = 1.602×10^(-19) C

For GaN ($m^* = 0.20\ m_0,\ \varepsilon_r = 9.0$):

C_{GaN} = $(4\pi \times \sqrt{(2 \times 0.20 \times 9.109\times10^{-31} \times 8.854\times10^{-12} \times 9.0)}) / (6.626\times10^{-34})$
= 1.44×10^{9} $eV^{-1}\cdot cm^{3/2}$

Normalized values (relative to GaN = 1.00):
- Si ($m^* = 1.08,\ \varepsilon_r = 11.7$): C = 1.15
- SiC ($m^* = 0.40,\ \varepsilon_r = 10.0$): C = 1.26

- GaN (m^*= 0.20, ε_r = 9.0): C = 1.00 (reference)
- Ga_2O_3 (m^*= 0.28, ε_r = 10.0): C = 1.18
- AlN (m^*= 0.30, ε_r = 8.5): C = 1.13
- Diamond (m^*= 0.70, ε_r = 5.5): C = 0.66

C varies by factor of 1.9× across materials (0.66 to 1.26).

S1.2 Barrier Height Scaling with Bandgap

Linear regression: φ_{Bn} = (0.28 ± 0.04) × E_g + (0.12 ± 0.15) eV
R^2 = 0.72 (moderate correlation, reflecting pinning strength variations)

S1.3 Achievable Doping vs Bandgap

- Dopant ionization energy scaling: E_d ≈ 0.02-0.05 × E_g
- DX center formation above critical Al composition in nitrides
- Process limitations (activation efficiency)

Empirical fit: $\log_{10}(N_{D,\max}/\text{cm}^{-3})$ = 21.5 - 0.7 × (E_g/eV)

S1.4 Combined Scaling Prediction

For two materials with ΔE_g = 1.0 eV:

$\Delta\varphi_{Bn}$ ≈ 0.28 eV (from S1.2)
$N_{D,1}/N_{D,2}$ ≈ $10^{-0.7}$ ≈ 0.2 (from S1.3)

$$\rho_{c,2}/\rho_{c,1} = \exp[C_2\varphi_{Bn,2}/\sqrt{N_{D,2}} - C_1\varphi_{Bn,1}/\sqrt{N_{D,1}}]$$

Result: Predicted increase = 0.7-1.2 orders of magnitude per eV
Measured: 0.8-1.0 orders per eV (from linear fit, Fig. 4)
Agreement within ±0.5 orders (scatter from C variations, pinning differences)

Conclusion: The 0.8-1.0 eV/decade scaling emerges from field emission physics combined with empirical E_g-dependent trends in φ_{Bn} and $N_{D,\max}$.

S2. Comparative Analysis

The empirical scaling holds because:

1. Barrier height dominates: φ_{Bn} scales approximately as 0.3–0.4 × E_g due to Fermi-level pinning (S ≈ 0.1–0.6), giving φ_{Bn} = 1.0 eV (SiC) → 1.4 eV (GaN) → 1.9 eV (Ga_2O_3) → 2.4 eV (AlN).
2. Exponential amplification: Even modest C variations become secondary when exponentially amplified. For example, comparing GaN to AlN at N_D = 10^{19} cm^{-3}:
   - Barrier effect: $\exp(1.9/\sqrt{N_D})$ / $\exp(1.4/\sqrt{N_D})$ ≈ 50× (dominant)
   - C variation: (1.01/0.85) ≈ 1.2× (secondary)

3. Doping compensation: Across the materials, achievable N_D also decreases (10^{20} for GaN → 10^{18} for AlN), which acts in the same direction as E_g increase.

The "~1 order per 0.8–1.0 eV" is therefore a phenomenological fit to the combined effects of barrier height scaling and achievable doping, with C variations contributing ~20–30% scatter around the trend line.

Table S1. Cross-material scaling of minimum achieved specific contact resistivity with bandgap.

**Material**	E_g **(eV)**	m^*/m_0	ε_r	C **(norm)**	φ_{Bn} **(eV)**	**Best** N_D **(cm⁻³)**	**Predicted** ρ_c	**Measured** ρ_c
SiC	3.26	0.40	10.0	1.26	1.0	2×10^{19}	$\sim10^{-6}$	8×10^{-7}
GaN	3.40	0.20	9.0	0.85	1.1	$>10^{20}$	$\sim10^{-8}$	3×10^{-8}
Ga_2O_3	4.80	0.28	10.0	1.06	1.9	$\sim10^{20}$	$\sim10^{-7}$	8×10^{-7}
AlN	6.10	0.30	8.5	1.01	2.4	$\sim10^{18}$	$\sim10^{-4}$	8×10^{-5}
